# A Novel Rat Infant Model of Medial Temporal Lobe Epilepsy Reveals New Insight into the Molecular Biology and Epileptogenesis in the Developing Brain

**DOI:** 10.1155/2024/9946769

**Published:** 2024-07-25

**Authors:** Carola Wormuth, Anna Papazoglou, Christina Henseler, Dan Ehninger, Karl Broich, Britta Haenisch, Jürgen Hescheler, Rüdiger Köhling, Marco Weiergräber

**Affiliations:** ^1^ Experimental Neuropsychopharmacology Federal Institute for Drugs and Medical Devices (Bundesinstitut für Arzneimittel und Medizinprodukte, BfArM), Kurt-Georg-Kiesinger-Allee 3, 53175, Bonn, Germany; ^2^ Translational Biogerontology German Center for Neurodegenerative Diseases (Deutsches Zentrum für Neurodegenerative Erkrankungen, DZNE), Sigmund-Freud-Str. 27, 53127, Bonn, Germany; ^3^ Federal Institute for Drugs and Medical Devices (Bundesinstitut für Arzneimittel und Medizinprodukte, BfArM), Kurt-Georg-Kiesinger-Allee 3, 53175, Bonn, Germany; ^4^ German Center for Neurodegenerative Diseases (Deutsches Zentrum für Neurodegenerative Erkrankungen, DZNE), Venusberg-Campus 1/99, 53127, Bonn, Germany; ^5^ Center for Translational Medicine Medical Faculty University of Bonn, Bonn, Germany; ^6^ Institute of Neurophysiology University of Cologne, Faculty of Medicine, Robert-Koch-Str. 39, 50931, Cologne, Germany; ^7^ Center of Physiology and Pathophysiology University of Cologne, Faculty of Medicine, Robert-Koch-Str. 39, 50931, Cologne, Germany; ^8^ Oscar Langendorff Institute of Physiology University of Rostock, Gertrudenstraße 9, 18057, Rostock, Germany

## Abstract

Although several adult rat models of medial temporal lobe epilepsy (mTLE) have been described in detail, our knowledge of mTLE epileptogenesis in infant rats is limited. Here, we present a novel infant rat model of mTLE (InfRPil-mTLE) based on a repetitive, triphasic injection regimen consisting of low-dose pilocarpine administrations (180 mg/kg. i.p.) on days 9, 11, and 15 *post partum* (pp). The model had a survival rate of >80% and exhibited characteristic spontaneous recurrent electrographic seizures (SRES) in both the hippocampus and cortex that persisted into adulthood. Using implantable video-EEG radiotelemetry, we quantified a complex set of seizure parameters that demonstrated the induction of chronic electroencephalographic seizure activity in our InfRPil-mTLE model, which predominated during the dark cycle. We further analyzed selected candidate genes potentially relevant to epileptogenesis using a RT-qPCR approach. Several candidates, such as the low-voltage-activated Ca^2+^ channel Ca_v_3.2 and the auxiliary subunits *β*_1_ and *β*_2_, which were previously reported to be upregulated in the hippocampus of the adult pilocarpine mTLE model, were found to be downregulated (together with Ca_v_2.1, Ca_v_2.3, M_1_, and M_3_) in the hippocampus and cortex of our InfRPil-mTLE model. From a translational point of view, our model could serve as a blueprint for childhood epileptic disorders and further contribute to antiepileptic drug research and development in the future.

## 1. Introduction

Numerous approaches to generate mesial temporal lobe epilepsy (mTLE) models have been described in the literature. mTLE can be induced either electrically or pharmacologically by injection of pilocarpine or kainic acid (KA) [1, 2]. The pilocarpine-induced *status epilepticus* (SE) rat model has emerged as the most widely used approach for mTLE models and is close to an ideal translational model in terms of isomorphism, homology, and predictability [3, 4]. The many variations make it difficult to claim a standard protocol for the adult rat pilocarpine mTLE model. However, standard parameters can be defined, starting with blockade of peripheral cholinergic side effects by administration of, e.g. atropine methylbromide (5 mg/kg i.p.) or methylscopolamine (1–2 mg/kg i.p.) [3, 4, 5, 6, 7, 8, 9]. Around 20 min later, single-dose pilocarpine administration (320–360, 380, or 400 mg/kg, i.p.) is carried out to evoke SE [3, 4, 5, 6, 7, 8, 9]. Approximately 60% of adult rats treated with pilocarpine successfully develop SE [10]. SE spontaneously resolves 5–6 hr after pilocarpine administration, and the animals enter a postictal coma. The latter can last 1–2 days if SE is not terminated pharmacologically [1, 11]. Most investigators induce SE with tonic–clonic generalized seizures lasting 90, 120, and >120 min [5, 6, 7, 8, 9]. This convulsive seizure activity typically begins within 10–30 min of the single-dose pilocarpine injection. Finally, an anticonvulsant is injected, such as diazepam (2–4 treatments, 10 mg/kg each, i.p., at 2–3 hr intervals if necessary) or other benzodiazepines and/or barbiturates (50 mg/kg pentobarbital i.p.) to terminate SE after 2 or more hours. Notably, inadequate suppression of SE usually results in high mortality [1, 12]. After a seizure-free period, i.e. the latent period, spontaneous recurrent seizures (SRS, chronic phase, chronic epileptic condition) occur. These eventually constitute the mTLE phenotype [8, 10]. Mortality rates of 30%–40% have been reported in male Wistar rats exposed to 300–400 mg/kg pilocarpine (i.p.) [1, 2, 3, 4, 9, 10, 11, 13]. However, mortality rates vary widely in different adult rat models of mTLE, ranging from 5% to 55% [1, 2, 3, 9, 10, 11, 13].

Interestingly, adult rats that did not exhibit convulsive SE following pilocarpine injection were capable of developing spontaneous seizures later in life but with a longer period of remission and reduced seizure frequency compared to animals with previous SE [14]. Although the pilocarpine-induced mTLE model is widely used to study seizure activity in adult rats [1, 3, 4, 11, 15], previous attempts to provide a neonatal, infant, or juvenile pilocarpine-induced mTLE model that is reliable, easily reproducible, and exhibits chronic epilepsy into adulthood have been challenging [16, 17, 18, 19, 20, 21]. Indeed, neuronal damage during a critical time window of early brain development can lead to devastating structural and functional changes later in adulthood [22, 23]. Such alterations are most evident in the hippocampus and, after a period of quiescence, are exacerbated by spontaneous seizure activity (29, 30). Particularly in neonatal, infant, and juvenile rats, the ictogenic/epileptogenic mechanisms underlying the pathophysiological reorganization of the immature brain from a normal to a chronic epileptic state are still unclear.

There is an urgent need for neonatal, infant, and juvenile mTLE models because of the numerous physiobiochemical differences between the immature and mature brain [24, 25]. Several attempts to develop such mTLE models were described in literature [16]. However, these models turned out to be insufficiently affected by the pilocarpine injections. Increased age (days 12–21 pp) seemed necessary to observe SE with a high lethality of 50% [16]. Other studies suggested that SRS can only be induced by pilocarpine injection in rats over 18 days of age with a rather low efficiency (22%) and an unsatisfying survival rate of 38% (at 18–24 days of age) [20].

Although a single injection of pilocarpine is effective in inducing SE and SRS in the adult rat model of mTLE [3, 4, 11], it was not efficient in infant rats [19]. Infant rats (day 12 pp) were injected with pilocarpine to induce SE, and 3 months later, only 25% of the rats exhibited SRS [19]. Long-term monitoring further revealed that 3 months after pilocarpine-induced SE in 12 days old rats, only 23% of the experimental animals displayed hippocampal neurodegeneration and volume reduction [26].

As single injections of pilocarpine appeared to be ineffective in inducing mTLE in infant rats, a sequential multiple injection approach was proposed using 380 mg/kg i.p. pilocarpine in infant rats on days 7, 8, and 9 pp [17, 18]. This injection regimen resulted in acute and chronic EEG changes and cognitive/behavioral deficits but only minor histological signs of neurodegeneration [17, 18]. Interestingly, all infant rats (male Wistar pups) that received the high pilocarpine dose of 380 mg/kg i.p. on 3 consecutive days were reported to survive the procedure [17, 18]. EEG recordings at days 30, 60, and 90 pp revealed episodes of (poly)spikes, also simultaneously in both the hippocampus and cortex. The long-term effects of three consecutive episodes of SE were further associated with increased spontaneous exploration, learning impairment, and limited anxiety at day 60 pp [17, 18]. However, in two other studies, the same model exhibited a lethality of 8.9% (with most animals (6.7%) dying after the first session) and 16%, respectively [27, 28]. The surviving animals again did not exhibit behavioral alterations associated with epileptic behavior. Interestingly, structural investigations revealed altered spatial patterns of neocortical interneurons, and, i.a., altered expression of hippocampal NMDA and AMPA receptors [27].

Here, we present a novel, highly efficient, triphasic low-dose pilocarpine injection regimen with diazepam-mediated SE termination and a 1–3 day(s) recovery period following each injection. This approach results in an infant rat model of mTLE (InfRPilo-mTLE) with spontaneous recurrent electroencephalographic seizures (SRES) and a high final survival rate. We investigated in detail the individual seizure parameters and their circadian dependence and performed molecular biology studies to investigate potential mechanisms involved in early ictogenesis. During the establishment of the new InfRPil-mTLE model, the relevance of age/body weight/developmental stage, pilocarpine dosage and number of consecutive injections, and diazepam dosage and method of application, as well as the duration of SE, were investigated. Our InfRPilo-mTLE model provides novel insights into the long-term impact of neuronal damage during early brain development. This may be of great value for future drug discovery and development as well as pharmacovigilance studies of antiepileptic drugs (AEDs) in infants and young children.

## 2. Materials and Methods

### 2.1. Study Animals

Wistar rat dams (Wistar rat IGS, Crl:WI) were delivered with 10 male pups each, at day 5 pp, from Charles River (Sulzfeld, Germany). The day of birth was designated as day 0 pp. We used only males in our study because the estrous cycle can have a strong influence on epileptogenesis and can significantly modulate the pharmacodynamics and pharmacokinetics of experimental drugs [29]. Each litter was housed together with the dams in clear Macrolon cages type III with *ad libitum* access to drinking water and standard foot pellets. Using ventilated cabinets (UniProtect cabinet, Zoonlab, Germany), rats were maintained at a standard ambient temperature of 22 ± 2°C, 50%–60% relative humidity, and a conventional 12 hr dark/light cycle with dark onset at 05 : 00 p.m. Animals were strictly adapted to this circadian pattern prior to subsequent experimentation.

All animal procedures were performed according to the guidelines of the German Council on Animal Care, and all protocols were approved by the Local Institutional and National Committee on Animal Care (State Office of North Rhine-Westphalia; Department of Nature, Environment and Consumerism; Landesamt für Natur, Umwelt und Verbraucherschutz; LANUV, NRW, Germany, AZ 84-02.04.2011.A297). The authors certify that all animal experimentation was carried out in accordance with the National Institute of Health Guide for the Care and Use of Laboratory Animals (NIH Publications No. 80-23) reviewed 1996, the UK Animals (Scientific Procedures) Act 1986 and associated guidelines, and the European Communities Council Directive of November 24, 1986 (86/609/EEC) and of September 22nd, 2010 (2010/63/EU). Specific effort was made to minimize the number of animals used and their suffering (3R strategy).

### 2.2. Pilocarpine Injection Regimen for a Novel Infant Rat Model of mTLE

In order to establish a novel infant rat model of mTLE, intensive pretesting for optimal pilocarpine and diazepam dosage finding, injection time points (based on age/weight relationship), and appropriate injection reiteration was carried out.

**Figure 1:**
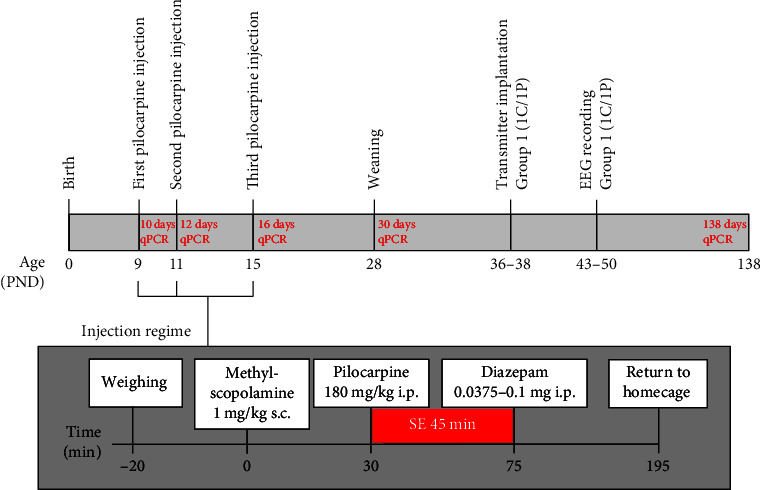
Schematic overview of the novel InfRPil-mTLE model using a triphasic pilocarpine injection regimen. At day 9 pp, experimental animals received the first injection regimen. Initially, pups were weighted, and methylscopolamine was administered subcutaneously. Thirty minutes later, pilocarpine was injected to the animals of the treatment group (P), whereas sham-treated control animals (C) received 0.9% NaCl. Pups were allowed to exhibit behavioral SE for a maximum duration of 45 min before convulsive seizure activity was terminated using ascending doses of diazepam. The diazepam dosage was always adapted to a minimum dose to terminate SE. Therefore, diazepam dosages were individually increased stepwise (0.025 mg steps), depending on the pups' body weight, SE severity, and individual responsiveness to diazepam. Sham-treated animals were administered 0.0375 mg diazepam at days 9 and 11 pp and 0.0625 mg at day 15 pp to mimic average diazepam dosages in the treatment group. Following diazepam administration, pups were monitored for additional 2 hr prior to return to their home cage/dam where they stayed until day 28 pp before being weaned. Pilocarpine-treated (P) and sham-treated control (C) rats were divided into different EEG telemetry subgroups based on their age at the time of radiofrequency transmitter implantation and subsequent long-term EEG recordings (see also Table 2). As early as day 43 pp, M1 motor cortex and hippocampal CA1 EEG recordings from group 1P displayed electroencephalographic seizure activity (see also Figure 2). Hippocampal CA1 and primary motor cortex tissue from nontransmitter implanted pilocarpine treated and untreated control animals were extirpated at days 10, 12, 16, 30, and 138 pp for qPCR analysis (hereinafter termed qPCR groups 10 days, 12 days, 16 days, 30 days, and 138 days, respectively; see also Table 1).

The pilocarpine-treated experimental animals (*verum* groups) received specific pilocarpine injection regimens outlined in Figure 1. To avoid circadian interdependence, the pilocarpine injection regimen was always administered between 9:00 a.m. and 12:00 a.m [30]. On day 9 pp, pups were separated from their dams 20 min prior to the treatment. At least one pup per litter remained with the dam to limit the risk of the dam rejecting the pups following the injection sequence. First, pups were weighed and marked (fur color-labeled; animal marker, Muramachi, Japan) and received a s.c. injection of methylscopolamine nitrate (1.13 ± 0.01 mg/kg, Sigma–Aldrich, Germany) 30 min prior to subsequent pilocarpine injection. Methylscopolamine serves to antagonize peripheral agonistic effects of pilocarpine on the muscarinic system [31, 32].

Next, pilocarpine hydrochloride (180.39 ± 1.87 mg/kg, Sigma–Aldrich, Germany) was administered i.p., and pups were closely monitored to determine the onset of a behavioral SE. Importantly, the pilocarpine dosage was adapted to the minimum dose able to induce a SE phenotype in the infant rats.

The pups were closely monitored and allowed 45 min to exhibit status-like motoric exacerbation before administration of diazepam (maximum final dosage ranging from 0.0375–0.1 mg i.p., Ratiopharm, Germany). This eventually terminated the tonic–clonic convulsive seizure activity. As preliminary testing revealed limited manageability of diazepam injection in rat pups, termination of SE was initiated with a low diazepam dosage of 0.0375 mg and increased stepwise (0.0250 mg), depending on the severity of SE and the age of the pups (up to 0.1 mg). This approach is based on the minimal dosage concept of diazepam administration described previously [1].

Throughout the entire injection procedure (Figure 1), cages with pups were placed upon a homeothermic heating blanket (ThermoLux®, Witte + Sutor, Germany) to maintain body core temperature. Following diazepam treatment, pups were allowed 2 hr to recover before being returned to their home cage and dam. Half of the home cage remained on the heating blanket to support body core temperature for 24 hr if necessary. The methylscopolamine–pilocarpine–diazepam injection sequence described above was repeated twice more, i.e., on days 11 and 15 pp (Figure 1). After completion of the triphasic injection regimen, pups were housed with their dams until weaning. Rats are normally weaned at day 21 pp; due to the developmental impairment upon pilocarpine treatment (Table 1), weaning in our experimental approach was extended to day 28 pp [19, 26].

It should be noted that sham-treated control pups only received methylscopolamine (1.13 ± 0.01 mg/kg, Sigma–Aldrich, Germany) and diazepam (0.0375 mg diazepam at days 9 and 11 pp and 0.0625 mg at day 15 pp). However, pilocarpine was substituted by 0.9% NaCl as vehicle (150 *µ*L at day 9 pp, 150 *µ*L at day 11 pp, and 250 *µ*L at day 15 pp). This sham-treatment concept has been described previously [32]. We classified our infant rat pilocarpine model of mTLE as InfRPilo-mTLE based on the working definitions of male rat postnatal development categories proposed by Bell [33]: neonatal, 0–6d; infant, 7–17d; juvenile, 18–41d; peripubertal, 42–55d; and late adolescent, 56–70d.

### 2.3. Radiofrequency Transmitter Implantation and Stereotaxic Electroencephalographic Electrode Placement

Pilocarpine-treated (P) and sham-treated control (C) animals were treated as described above (Figure 1). In order to record and monitor EEG seizure activity from both the hippocampal CA1 and primary motor cortex (M1), experimental animals were implanted with a radiofrequency transmitter and stratified into different experimental groups according to the age of implantation and the time of subsequent EEG recording. Rats from groups 1C (*n* = 12) and 1P (*n* = 11) were implanted at the age of 36–38 days and EEG recordings were performed on days 43–50 pp. Animals from groups 2C (*n* = 11) and 2P (*n* = 9) were implanted at the age of 57–59 days pp, and EEGs recorded on days 64–70 pp. Finally, rats of groups 3C (*n* = 13) and 3P (*n* = 24) were implanted at the age of days 119–123 pp and recorded on days 127–134 pp and days 137–142 pp (Table 2).

Notably, the age-related stratification of experimental groups proved out to be mandatory, as the duration of optimal, high-quality EEG recordings of the telemetry system used here is normally limited to 4–8 weeks due to growth of the skull and ossification processes from the drilled holes [34, 35, 36, 37]. It was therefore necessary to define specific age-related homogenous implantation groups to ensure reliable EEG recordings over the entire experimental period and to be able to record EEGs under comparable conditions.

For EEG radiofrequency transmitter implantation, rats were anesthetized with inhalational isoflurane (Isoflurane Baxter, 100% V/V) at a final concentration of 2%–3% isoflurane together with carbogen (5% CO_2_ and 95% O_2_, 0.5–1 L/min). The volatile anesthetic isoflurane was applied using a Matrix TM VIP 3000 Calibrated Vaporizer and a scavenger system (both from Harvard Apparatus; USA) [34, 36]. Endotracheal intubation and ventilation were not performed. Subsequently, a radiofrequency transmitter (TL11M2-F20-EET, 2-channel transmitter, weight 3.9 g, volume 1.9 ccm, input voltage range ±1.25 mV, and channel bandwidth 1–50 Hz; Data Science International (DSI, Harvard Bioscience Inc., USA) was implanted into a s.c. pouch on the back of the animal [34, 36, 37]. Using a computerized 3D stereotaxic device attached to a rat stereotaxic frame (Neurostar, Germany), the differential epidural electrode of transmitter channel 1 targeting the primary motor cortex (M1) and the differential intracerebral electrode of channel 2 targeting the hippocampal CA1 region were positioned according to the stereotaxic coordinates presented in Table 3 [38, 41]. The reference electrodes for both EEG channels were placed on the cerebellar cortex (for stereotaxic coordinates, see Table 3), as the latter serves as a rather silent electroencephalographic region [34, 36, 38, 39]. For a two- and three-dimensional illustration of the electrode placement, see also *Supplementary figure 1*. Notably, stereotaxic coordinates in literature for the rat brain are predominantly based on male Wistar rats with weights ranging from 270 to 310 g, with claimed validity in the wider range of 250–350 g [39]. These coordinates were applied for the radiotelemetry groups 2C/2P and 3C/3P, whereas for groups 1C/1P, stereotaxic coordinates were weight-adjusted by 80% of the reference (Table 3).

Note that the longitudinal temporal characteristics of our EEG studies in juvenile and adolescent, rapidly developing rat brains, pose a critical challenge for radiotelemetric long-term EEG recordings. The coordinates chosen here guarantee recordings from the region of interest (CA1 and M1) during the individual time windows of investigation for the individual EEG study groups. Nevertheless, slight changes in electrode positioning are inevitable during the course of the experiment and the developmental growth of rat brain and skull [42]. Importantly, proper electrode placement in our experimental animals was confirmed histologically *post mortem*.

After stereotaxic insertion, the electrodes were fixed on the skull using glass ionomer cement (Kent Dental®, Kent Express, UK), and the scalp was closed using over and over sutures (Sabafit, 6-0, SABANA Medizinbedarf, Wiesbaden, Germany). To avoid hypothermia, the animals were kept warm throughout the surgical procedure using a homeothermic heating blanket (ThermoLux®, Witte + Sutor, Murrhardt, Germany). Further details on transmitter implantation, positioning of brain electrodes and cranial fixation can be found in our previous publications [34, 36, 37]. For peri- and postoperative pain management, animals were administered carprofen (5 mg/kg s.c., Rimadyl, Parke-Davis/Pfizer, Germany) prior to surgery as well as once per day for 2 days postsurgery. Implanted rats were housed individually in single cages to avoid interindividual stress and wound manipulations. Supportive care was provided to the rats as required to facilitate recovery (s.c. administration of Ringer solution to substitute volume, control of body temperature, and temperature support if necessary). In our experimental setting, all animals of EEG telemetry groups 1C, 2C, 3C, 1P, 2P, and 3P survived the implantation procedure and the subsequent recording periods.

### 2.4. Radiotelemetric EEG Recordings and Data Acquisition

Animals were allowed to recover for 5–7 days postsurgery [34, 36]. Simultaneous long-term video-EEG recordings from the hippocampus (CA1 area) and the primary motor cortex (M1) were then performed for 5–8 days. As outlined above, pilocarpine-treated (P) and sham-treated control (C) rats were divided into six different EEG telemetry groups (1P−3P and 1C−3C) based on their age at radiofrequency transmitter implantation and subsequent EEG recording (see Table 2). Due to the larger simple size of group 3P, simultaneous recording of all these animals was not possible for technical reasons. It was therefore necessary to determine two distinct time periods for long-term EEG recordings (i.e., days 127–134 and 137–142 pp) for groups 3P and 3C in order to collect data from all animals. Importantly, the extended duration of recordings is not known to affect seizure results at this advanced age.

All EEG recordings were performed using Dataquest ART 4.35 Software (DSI, Harvard Bioscience Inc., USA) at a virtual sampling rate of 1,000 Hz with no *a priori* filter cutoff. Note that the TL11M2-F20-EET transmitter bandwidth (B) is 50 Hz with a nominal sampling rate (*f*) of 250 Hz, (*f* = 5 X B). Thus, based on the Nyquist–Shannon theorem, the radiofrequency transmitter used in our study can only reliably record frequencies up to 125 Hz [43].

### 2.5. Electroencephalographic Seizure Analysis

Electroencephalographic seizure analysis was performed using the Neuroscore® 3.2 automated seizure detection module (DSI, Harvard Bioscience Inc., USA). For all experimental animals, the EEG baseline amplitude was determined individually for both the hippocampal CA1 and cortical M1 EEG recordings. For a seizure-like event to be scored, the amplitude threshold of ictal discharges (*μ*V) was set to 1.5x above the baseline. Further parameters for automated seizure detection included maximum voltage, 1,000 *μ*V; minimum spike duration, 1 ms; maximum spike duration, 1,000 ms; and spike train parameters, minimum spike interval, 0.05 s; maximum spike interval, 1.5 s; maximum train duration, 1 s; train join interval, 1 s; and minimum number of spikes, 2. It should be noted that the Neuroscore® default seizure parameter settings mentioned above exhibit high sensitivity but impaired specificity, as discussed later. Importantly, in order to explore potential circadian interdependencies, electroencephalographic seizures were analyzed for the entire long-term recordings and additionally for both the dark (D) and light (L) cycles. The following seizure parameters were calculated and averaged over a 12 hr period: (i) number of spike trains, (ii) total spike train duration (min), (iii) spike train coverage (%), (iv) average spike train duration (s), (v) maximum spike train duration (s), (vi) average spike number/spike train, and (vii) number of single spikes and single spike coverage (%). Video recordings of experimental animals during recording were used to check for potential artifacts, e.g., eating, grooming, and other voluntary movements, that could mimic or trigger ictal-like discharges in the EEG. Analysis between groups of pilocarpine mTLE rats and sham-treated controls was performed with unpaired *t*-tests (1C vs. 1P, 2C vs. 2P, and 3C vs. 3P). Group differences and developmental effects between the different age-related treatment and control study groups were analyzed using two-way ANOVA followed by Tukey's multiple comparison test. All analyses were performed using GraphPad Prism 6.04 (GraphPad Software, La Jolla California USA). Results are displayed as mean ± SEM.

### 2.6. Validation of EEG Electrode Placement

After EEG recordings, animals were deeply anesthetised with ketamine/xylazine (100/10 mg/kg, i.p.; Ketanest® S, 100 mg/ml, Pfizer; Rompun® 2%, Bayer Health; Germany). Subsequently, implanted rats underwent cardiac perfusion for 10 min using ice-cold phosphate-buffered saline (PBS, pH 7.4) followed by 4% paraformaldehyde (PFA) in PBS (pH 7.4) for another 10 min. Afterward, brains were extirpated, fixed for 2–4 hr in 4% PFA at RT, cryoprotected in 30% sucrose in PBS (pH 7.4), and stored at 4°C for at least 4 weeks until further processing [44]. Brains were frozen in Tissue-Tek (Sakura, Japan) on a stereotaxic block and cut into 40-*µ*m-thick coronal slices using a Leica CM3050 S cryostat (Leica Instruments, Germany). The slices were then mounted on Superfrost® Plus glass slides (Thermo Scientific, Germany), air dried, and stained using methylene blue (Sigma–Aldrich, Germany) to verify the correct electrode placement. As all implanted rats met strict EEG electrode placement criteria, no implanted and recorded animals were excluded from the analysis.

### 2.7. RNA Extraction from the Hippocampus and Motor Cortex

Several susceptibility genes in mTLE epileptogenesis were checked for potential transcriptional alterations using RT-qPCR. The latter included (i) different VGCCs (i.e., Ca_v_1.3, Ca_v_2.1, Ca_v_2.2, Ca_v_2.3, Ca_v_3.1, Ca_v_3.2, and Ca_v_3.3), (ii) VGCC auxiliary subunits (*β*_1–3_ and *α*_2_*δ*), and (iii) muscarinic receptors (M_1_, M_3_, and M_5_). Total RNA was extracted from the hippocampus and motor cortex (100 mg) using RNeasy Lipid Tissue Mini Kit including a DNase digest (Qiagen, Germany). A total of five different age groups were used (including pilocarpine-treated and pilocarpine-untreated animals, *n* = 3–4 per group, all ♂) (Figure 1, Table 1): the pilocarpine-treated group 10d received a single pilocarpine injection containing methylscopolamine (1.10 ± 0.01 mg/kg, s.c.), pilocarpine (180.54 ± 1.26 mg/kg, i.p.), and diazepam (0.0375–0.1 mg, i.p.) at day 9 pp, and animals were sacrificed for cortical and hippocampal RNA extraction at day 10 pp. In the same manner, the pilocarpine-treated group 12d received two pilocarpine injections at days 9 and 11 pp, and pups were sacrificed at day 12 pp; the pilocarpine-treated group 16d received three pilocarpine injections at days 9, 11, and 15 pp, and pups were sacrificed at day 16 pp; the pilocarpine-treated group 30d and 138d, similar to group 16 d, also received all three pilocarpine injections, and pups were sacrificed for RNA extraction at days 30 and 138 pp, respectively (Figure 1; Table 1). Untreated control groups did not receive pilocarpine injections. In addition, total hippocampal and motor cortex RNA was extracted from three untreated adult rats (aged 86d; body weight 439.9 ± 20.8 g, all ♀) using RNeasy Lipid Tissue Mini Kit including DNase digest (Qiagen, Germany). The latter served as a calibrator to reduce interrun variation. Notably, all animals used for RT-qPCR (hereinafter referred to as groups 10d, 12d, 16d, 30d, and 138d) were not included in any EEG radiotelemetry studies (referred to as groups 1C−3C and 1P−3P).

### 2.8. Reverse Transcriptase Quantitative Real-Time PCR (RT-qPCR)

Isolated RNA was reversely transcribed into cDNA in a two-step RT-qPCR approach using both anchored oligo (dT)_18_ and random hexamer primers (Transcriptor First Strand cDNA Synthesis Kit, Roche, Germany) (Table 4). qPCR was performed using a Light Cycler 480 System (Roche, Germany). For this, the following protocol was applied: 95°C (10 min, preincubation step) and 35 cycles with each cycle being composed of a denaturation step (95°C, 10 s) an annealing step (60°C, 20 s) and an extension step (72°C, 30 s). The specificity of amplification was controlled by melting curve analysis. In addition, deionized, nuclease-free water (no template probe) and total RNA samples (no reverse transcriptase probe) were used as negative controls to exclude false-positive results. Analysis of RT-qPCR data were performed according to Hellemans et al. [46], with data normalized to both the internal control gene (hypoxanthine–guanine–phosphoribosyltransferase (HPRT)) and a calibrator. This calculation is based on the model of the *ΔΔ*Ct (cycle threshold) approach [47], supporting the use of gene specific amplification efficiencies and normalization with reference gene. Calculation of the fold changes (FC) and the calibrated normalized relative quantities (CNRQ) for each individual gene as well as the statistical analysis (Mann–Whitney test and one-way ANOVA test) was carried out using qBase^+^ software (Biogazelle, Belgium).

## 3. Results

### 3.1. Establishment of the Novel InfRPil-mTLE Model

To establish the novel InfRPil-mTLE model, several parameters, such as age/body weight/developmental stage, pilocarpine and diazepam dosages, and number of consecutive injection regimens, methods of drug application (s.c. vs. i.p.), and the maximum duration of induced seizure activity/SE, were tested and optimized. Based on the initial correlation studies between survival rate and effectiveness in establishing long-term epileptic activity originating from early pilot experiments, the most effective injection regimen was selected and used to establish the novel InfRPil-mTLE model as illustrated in Figure 1. Our novel InfRPil-mTLE model was then characterized on both electrophysiological and molecular levels.

In the final protocol, pups received three consecutive pilocarpine injections (180.39 ± 1.87 mg/kg, i.p.) at days 9, 11, and 15 pp to induce seizure activity (potentially escalating to SE), as described in Figure 1. Thirty minutes prior to pilocarpine injection, methylscopolamine (1.13 ± 0.01 mg/kg, s.c.) was administered to antagonize peripheral muscarinic action in the experimental animals. The pilocarpine-induced seizure activity/SE was characterized behaviorally by motoric exacerbation including one or more of the following phenomena (see Racine scale): (i) oral automatisms, (ii) excessive scratching, (iii) head bobbing, (iv) shaking, (v) forelimb clonus, (vi) rearing and falling, (vii) loss of upright posture, and eventually, (viii) tonic–clonic seizures [32, 48]. Notably, the behavioral characteristics of SE were largely inhomogenous among the experimental rat pups. Forty-five minutes *post* pilocarpine injection, diazepam (0.0375–0.1 mg, i.p.) was administered stepwise in 0.025 mg doses. Final diazepam dose was increased until seizure activity ceased (Figure 1). Sham-treated control animals received the same injection regimen as pilocarpine-treated rats. However, pilocarpine was substituted by 0.9% NaCl, and 45 min post injection, rats were administered a single diazepam dosage based on their age (0.0375 mg for days 9 and 11 pp; 0.0625 mg for day 15 pp).

It is noteworthy that the highly efficient triphasic injection regimen for mTLE in infant rats established here differs significantly from the well-described adult pilocarpine mTLE model protocols. In the latter, adult rats are routinely administered a single dose of pilocarpine (e.g., 300–380 mg/kg, i.p.), which is sufficient to induce SE in most studies [3, 6, 49, 50, 51]. However, in infant rats up to day 15 pp, a single, high-dose pilocarpine injection does not induce SE and/or long-term ictogenic/epileptogenic effects [16, 17, 18, 19, 20, 52]. In our pilot experiments, administration of a single pilocarpine dosage (i.e., 340 mg/kg, i.p.) in infant Wistar rats at days 8 or 16 pp resulted in a survival rate of 33% and 25%, respectively, with all animals exhibiting seizure activity (data not shown). The mortality rates associated with a single high-dose injection of pilocarpine to establish a chronic mTLE model in infant rats, similar to the doses used in the adult model, thus proved to be unacceptably high. In contrast, using a single pilocarpine injection of only 170 mg/kg (i.p.), i.e., half the amount of the initial dose, in 10-day-old rat pups, resulted in 100% survival rate. In addition, all animals exhibited isolated motor seizures and/or SE (data not shown). Under the same experimental conditions, similar results were observed with young rats at day 17 pp. Unfortunately, pilocarpine-induced acute seizure activity/SE at days 10 or 17 pp did not lead to chronic spontaneous seizures later in life, when animals were monitored for weeks to months (data not shown). Based on the literature [3, 16, 17, 18, 19, 20, 52] and our own pretesting, we concluded that a repetitive (triphasic) application of a moderate pilocarpine dosage would be more efficient in establishing a chronic mTLE model originating from infant rats. This approach was based on the assumption/observation that the infant rat brain is less susceptible to single pharmacologically induced excitotoxic events. Instead, a multiphasic, reiterating injection regimen was required to counteract the high regenerative capacity in the infant brain and to trigger an effective kindling process [53, 54].

Importantly, we also observed age-/weight-specific differences in the dosing. High single diazepam doses (e.g., 20 mg/kg, i.p.), which are frequently used in adult models of mTLE to terminate SE [6, 50], were in most cases lethal for the infant rats, even if they survived SE. In contrast, lower single diazepam dosages (e.g., 8 mg/kg i.p. or less) were generally well tolerated. Pilot studies using lower single dosages of diazepam (i.e., 6 and 4 mg/kg, i.p.) after surviving 45 min of seizure activity/SE exhibited 100% survival rate at days 10 and 17 pp in infant rats (data not shown). In addition, we observed that diazepam was much better tolerated by the rat pups when it was administrated stepwise in doses of 0.025 mg. The intervals between diazepam doses were individually adjusted based on the effectiveness to supress seizure activity.

Our novel triphasic pilocarpine injection regimen for the InfRPil-mTLE model described here is characterized by an overall survival rate of 80.3% following pilocarpine-induced seizure activity/SE and diazepam administration. The latter rate is similar to or even higher than that reported in the literature for pilocarpine-induced SE and mTLE generation in immature rat brains [26, 55, 56, 57]. The established adult rat models of mTLE normally exhibit only 30%–55% survival rates depending of the applied pilocarpine dosages [15].

### 3.2. Developmental Impairment in the InfRPil-mTLE Model

To gain insight into the potential developmental alterations in our model, we analyzed the body weight of pilocarpine-treated and pilocarpine-untreated rats. On day 10 pp, i.e., 1 day after the first pilocarpine injection on day 9 pp (group 10d; see Figure 1, Table 1), pilocarpine-treated animals exhibited a significant reduction in body weight compared to untreated controls (17.3 ± 0.4 g vs. 25.2 ± 0.8 g, *n* = 4, *p* < 0.0001; Table 1), indicating a developmental impairment upon seizure induction. This effect was also observed for the 12 and 16d experimental groups when comparing treated with untreated rats (group 12d, 24.5 ± 2.0 g vs. 31.4 ± 0.6 g, *n* = 4, *p*=0.016; group 16d, 36.9 ± 1.7 g vs. 44.8 ± 1.2 g, *n* = 4, *p*=0.001, Table 1). At a later stage (groups 30 and 138d), body weights did no longer differ significantly between pilocarpine-treated and untreated rats (group 30d, 104.2 ± 6.4 g vs. 109.5 ± 5.9 g, *n* = 4, *p*=0.559; group 138d, 588.6 ± 5.9 g vs. 560.6 ± 6.6 g, *n* = 4, *p*=0.076; Table 1). In conclusion, body weight gain was delayed during the course of the triphasic injection regimen (groups 10, 12, and 16d) used to establish the novel InfRPil-mTLE model (for graphical representation see also *Supplementary figure 2*). However, this effect disappears at later developmental stages (groups 30d and 138d). Notably, the reduction of weight in infant rats post pilocarpine injection in our model is consistent with previous reports of seizure induced developmental impairment and recovery of body weight later on [19, 26].

### 3.3. Qualitative Electroencephalographic Seizure Characteristics in the InfRPil-mTLE Model

As the duration of high-quality radiotelemetric EEG recordings is limited to a maximum of about 8 weeks in our experimental environment, and due to the growth of the brain and cranium during early development, different age groups were defined to ensure continuous EEG data collection over the course of ~5 months. Pilocarpine- (P) and sham-treated controls (C) male rats are represented by different EEG telemetry groups (1P−3P and 1C−3C) based on the age of the animals at the time of radiofrequency transmitter implantation and consequently, the timing of EEG recording as given in Table 2.

Using a two-channel EEG radiofrequency transmitter (DSI, Harvard Bioscience Inc., USA), recordings from the intracerebral hippocampal CA1 and primary motor cortex M1 region were analyzed for electroencephalographic seizure activity for all groups outlined above (*Supplementary figure 1*).

**Figure 2:**
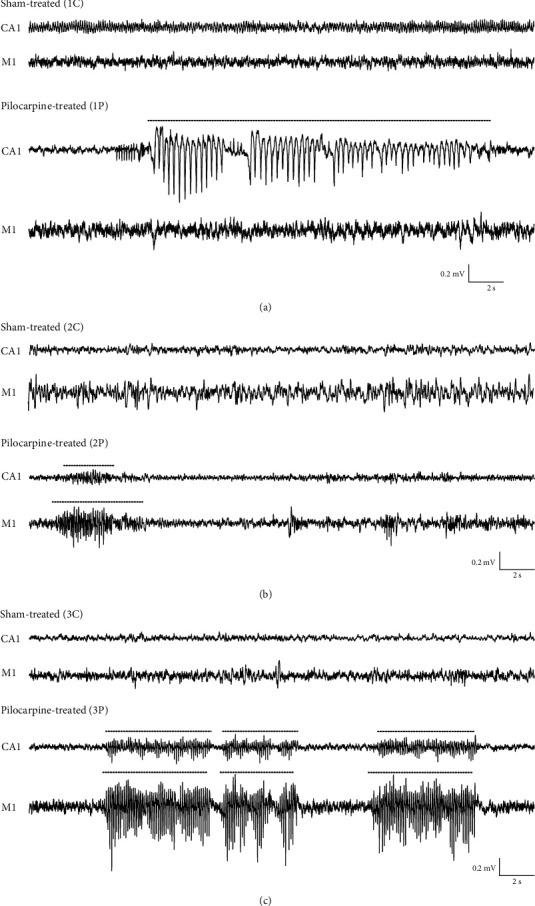
Hippocampal CA1 and cortical M1 EEG recordings from sham-treated control and pilocarpine-treated rats. Representative 30-s EEG traces of sham-treated controls (C) and pilocarpine-treated (P) rats (all **♂**) from the hippocampus (CA1) and the motor cortex (M1). Pilocarpine-treated rat pups were administered a triphasic injection regimen (see Figure 1) at days 9, 11, and 15 pp. Their sham-treated control littermates received 0.9% NaCl instead of pilocarpine: (a) Group 1C and 1P were implanted at days 36–38 pp and continuously recorded from days 43 to 50 pp. The EEG recording from a CA1 sham-treated control exhibits an example of theta oscillations which can be indicative of, e.g., alert immobility, exploratory behavior, or learning and memory processes. In M1 EEG recordings from sham-treated controls, higher frequencies, i.e., in the beta (>12 Hz) and gamma range (>30 Hz), predominate. In contrast to sham-treated control rats, prominent electroencephalographic seizure activity with fast high-amplitude continuous spiking, was detected in the hippocampal CA1 region of pilocarpine-treated animals (dashed line: spike train activity). Notably, different electroencephalographic seizure scenarios were observed, including isolated ictal discharges in CA1 (dashed line) or M1 recordings (not depicted here) or simultaneous ictal discharges in both deflections. (b) Groups 2C and 2P were implanted at days 57–59 pp and continuously recorded from days 64 to 70 pp. These traces display an example of coincident seizure activity (dashed lines) in CA1 and M1 deflections in the pilocarpine-treated group including subsequent isolated seizure activity in the M1 deflection. (c) Groups 3C and 3P were implanted at days 119–123 pp and continuously recorded from days 127–134 and 137–142 pp. Severe electroencephalographic seizures (dashed lines) were observed in both CA1 and M1 recordings of the pilocarpine-treated group 3P. Seizure activity is characterized by isolated spikes, spike waves, and polyspike waves. Spike trains exhibit a characteristic *crescendo*-*decrescendo* spindle-like pattern. In these segments, M1 electroencephalographic seizure activity seems to precede the occurrence of CA1 ictal discharges. Horizontal bar, 2 s; vertical bar, 0.2 mV.

Initial qualitative analysis of long-term electrocorticograms (M1) and electrohippocampograms (CA1) from our InfRPil-mTLE model revealed typical ictal discharges/epileptiform graphoelements, e.g., spikes, spike waves, and polyspike waves in all three pilocarpine-treated groups (1P−3P) (Figures 2(a), 2(b), and 2(c)) subjected to multiple SE during development. In contrast, sham-treated control animals (1C−3C) hardly ever exhibited ictal-like discharges. Apart from isolated ictal discharges, we also observed spindle-like spike/spike wave trains in InfRPil-mTLE animals characterized by seizure initiation, seizure continuation/perseveration, and termination. In sham-treated control animals, these ictal-like discharges were also significantly fewer and if present, could be related to behavioral phenomena, such as eating and grooming, as verified by the synchronized video/EEG recordings.

As outlined above, the behavioral seizure phenotype/severity of the SE period varied between individual rats/pilocarpine injections. Animals showing tonic convulsions during SE episodes seemed to be more susceptible to complex EEG abnormalities. However, a clear correlation between electrocorticographic and electrohippocampographic seizures on the one hand and behavioral manifestations of SE on the other hand could not be detected, similar to what has been reported previously [17, 18].

Importantly, the electrocorticographic (M1) and electrohippocampographic (CA1) epileptiform alterations in our InfRPil-mTLE model were not accompanied by major behavioral seizures. However, subtle, subconvulsive disturbances, i.e., behavioral arrest, and in some cases masticatory and orofacial stereotyped movements were observed. In rare cases, animals presented manifestations of spontaneous partial seizures, characterized by forelimb clonus and masticatory movements, concomitant with epileptiform discharges in both hippocampal and cortical recordings.

In many instances, electroencephalographic seizure activity was observed simultaneously in both CA1 and M1 recordings, but occasionally seizure activity was visible only in one area, either the hippocampus (CA1) or the motor cortex (M1) (Figure 2(a)). This is an important observation, indicating that the recording electrodes selectively detect extracellular potential changes at the insertion site and are not susceptible to EEG far-fields in this setting (sufficient spatial resolution).

### 3.4. Quantitative Electroencephalographic Seizure Characteristics in the InfRPil-mTLE Model

Seizure quantification of long-term radiotelemetric CA1 and M1 EEG recordings revealed major differences in seizure parameters between pilocarpine- and sham-treated control animals, with pilocarpine-treated animals showing significantly more seizure activity of longer duration in almost all parameters for both CA1 and the M1 recordings.

**Figure 3:**
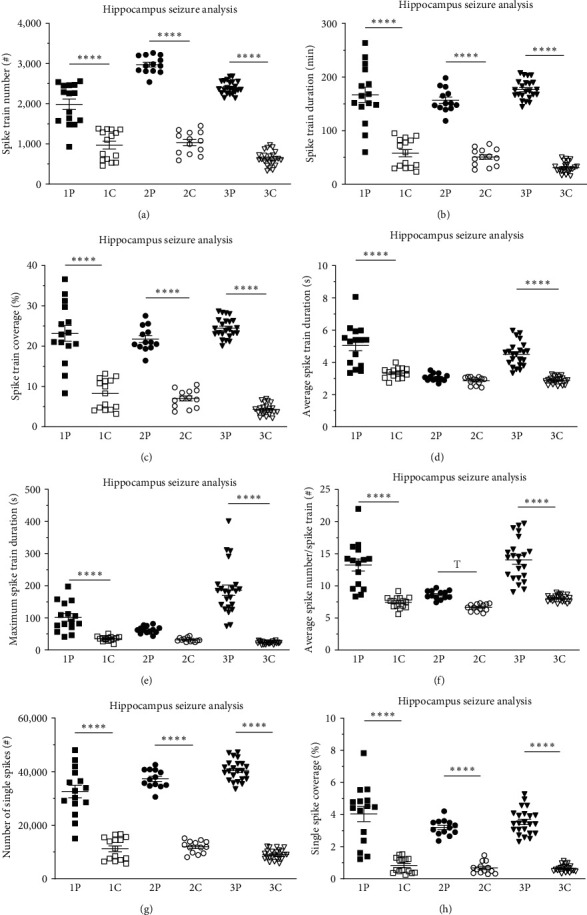
CA1 hippocampal electroencephalographic seizure analysis in the InfRPil-mTLE model. Analysis was carried out for pilocarpine-treated rat pups (P, black symbols) which received the triphasic injection regimen at days 9, 11, and 15 pp (see also Figure 1). Sham-treated control littermates (C, open symbols) were manipulated accordingly. However, pilocarpine injection was substituted by 0.9% NaCl. Pilocarpine- and sham-treated rats were divided into different EEG telemetry groups (1C and 1P, squares; 2C and 2P, circles; and 3C and 3P, triangles) based on the age of radiofrequency transmitter implantation and EEG recordings (see also Figure 1 and Table 2). The intracerebral, hippocampal EEG long-term recordings were analyzed for different seizure parameters, including (a) spike train number, (b) spike train duration (min), (c) spike train coverage (%), (d) average spike train duration (s), (e) maximum spike train duration (s), (f) average spike number per spike train, (g) total number of single spikes, and (h) single spike coverage (%). Data from experimental animals are displayed as scatter plots. Note that absolute values were averaged for a 12 hr period. In this analytical setting, potential circadian aspects are not included. For a light/dark cycle specific differentiation, see *Supplementary figure 4*. Importantly, all parameters analyzed revealed electroencephalographic seizures in the pilocarpine-treated groups (1P−3P), significantly higher compared to sham-treated animals (1C−3C). Residual spike activities in sham-treated rats might originate from confounding factors related to the deep electrode implantation and potential neuronal injury. Results are depicted as mean ± SEM;  ^*∗∗∗∗*^*p* < 0.0001. A statistical overview of the hippocampal seizure parameters within the pilocarpine-treated and the sham-treated control groups is depicted in *Supplementary table 7*.

A clear distinction between pilocarpine- and sham-treated control animals is observed in the hippocampal CA1 region in all groups for the number of spike trains (Figure 3(a)). All pilocarpine-treated groups (1P−3P) exhibited a significant increase compared to sham-treated control groups (1C−3C): 1P vs. 1C, 1984.8 ± 132.1 vs. 969.3 ± 95.6, *p* < 0.0001; 2P vs. 2C, 2972.3 ± 60.1 vs. 1032.6 ± 77.5, *p* < 0.0001; and 3P vs. 3C, 2402.9 ± 33.2 vs. 634.6 ± 37.4, *p* < 0.0001. Interestingly, the spike train number decreased significantly in a time-dependent manner for the sham-treated control groups (1C vs. 3C, 969.3 ± 95.6 vs. 634.6 ± 37.3, *p* < 0.01; *Supplementary table 7*). The latter observation might point to the fact that neuronal damage due to the implantation procedure might somewhat contribute to the spike generation in younger rats and less so in older ones.

A similar tendency was observed in the hippocampal CA1 region for the total spike train duration (Figure 3(b)). The spike train duration of pilocarpine-treated groups showed a significant increase in the InfRPil-mTLE model compared to sham-treated control groups: 1P vs. 1C, 166.9 ± 14.1 min vs. 58.0 ± 6.9 min, *p* < 0.0001; 2P vs. 2C, 156.8 ± 6.1 min vs. 50.8 ± 4.4 min, *p* < 0.0001; and 3P vs. 3C, 176.6 ± 3.8 min vs. 31.0 ± 2.1 min, *p* < 0.0001. Similar results were observed for spike train coverage: 1P vs. 1C, 23.2% ± 2.0% vs. 8.1% ± 1.0%, *p* < 0.0001; 2P vs. 2C, 21.8% ± 0.8% vs. 7.1% ± 0.6%, *p* < 0.0001; and 3P vs. 3C, 24.5% ± 0.5% vs. 4.3% ± 0.3%, *p* < 0.0001 (Figure 3(c)).

Additionally, seizure analysis revealed significant differences in average spike train durations and maximum spike train durations (Figures 3(d) and 3(e). The average spike train duration was increased in pilocarpine-treated groups 1P and 3P, compared to the corresponding sham-treated control (1C and 3C) animals: 1P vs. 1C, 5.0 ± 0.3 s vs. 3.3 ± 0.1 s, *p* < 0.0001, and 3P vs. 3C, 4.5 ± 0.2 s vs. 2.9 ± 0.1 s, *p* < 0.0001 (Figure 3(d)). In this context, the maximum spike train duration is the value of the longest spike train observed in each. As expected, in pilocarpine-treated animals, the maximum spike train duration is significantly longer compared to sham-treated controls: 1P vs. 1C, 101.4 ± 11.7 s vs. 35.0 ± 2.0 s, *p* < 0.0001, and 3P vs. 3C, 186.1 ± 16.2 s vs. 22.2 ± 0.9 s, *p* < 0.0001 (Figure 3(e)). There is also an age-dependent increase in maximum spike train duration: its duration in 3P is significantly longer (*p* < 0.0001) not only compared to 3C but also to the other two pilocarpine-treated groups (1P, 2P) (*Supplementary table 7*). These findings illustrate that with increasing age, the duration of electrographic seizures also increases, suggesting that the detrimental effect of pilocarpine-induced epilepsy in infant rats triggers further progressive neuronal damage and epileptiform activity during development. This increase in the maximum spike train duration also coincides with augmented levels of average number of spikes/spike train: 1P vs. 1C, 13.2 ± 0.9 vs. 7.4 ± 0.2, *p* < 0.0001, and 3P vs. 3C, 14.1 ± 0.7 vs. 8.0 ± 0.1, *p* < 0.0001; Figure 3(f)).

In accordance with the results of our spike train analysis (Figures 3(a), 3(b), 3(c), 3(d), 3(e), and 3(f)), the total number of single spikes (Figure 3(g)) is significantly increased in our InfRPil-mTLE model compared to sham-treated control (1P vs. 1C, 32575.0 ± 2310.0 vs. 11175.0 ± 1092.4, *p* < 0.0001; 2P vs. 2C, 37307.6 ± 953.0 vs. 11897.0 ± 627.5, *p* < 0.0001; and 3P vs. 3C, 40417.7 ± 797.6 vs. 9002.3 ± 398.5, *p* < 0.0001). Note that these seizure parameter values are averaged for a representative 12 hr period.

The number of single spikes in the sham-treated control animals actually decreases in 2C vs. 3C (11897.0 ± 627.5 vs. 9002.3 ± 398.5, *p* < 0.05; Figure 3(g)), in a similar fashion as the spike train number in Figure 3(a) (1C vs. 3C), indicating a developmental change in the adult rat brain and/or the effects of a healing process around the implantation side. Single spikes account for only a small percentage of the total EEG, as shown by the single spike coverage (Figure 3(h)), but with 4.5-fold increase in the InfRPil-mTLE model on average, compared to sham-treated controls: 1P vs. 1C, 4.0% ± 0.5% vs. 0.8% ± 0.1%, *p* < 0.0001; 2P vs. 2 C, 3.2% ± 0.1% vs. 0.7% ± 0.1%, *p* < 0.0001; and 3P vs. 3C, 3.5% ± 0.2% vs. 0.6% ± 0.0%, *p* < 0.0001). In general, the InfRPil-mTLE model exhibits a significant increase in nearly all parameters analyzed in the hippocampal CA1 region. This observation underscores the severe persistent brain injury induced by the triphasic pilocarpine injection regimen in the infant developmental stage, leading to an electroencephalographic epileptic phenotype as seen in our EEG recordings. The alleged minor ictal-like discharges in the sham-treated control groups are probably due to possible neuronal damage at the electrode implantation side [58]. It is clear that the same unavoidable methodological limitation might be present in the pilocarpine-treated groups. Therefore, we also normalized seizure parameters in the hippocampus of our InfRPil-mTLE model, illustrating relative values of the pilocarpine-treated groups for each seizure parameter after setting the sham-treated control group values to 1 (*Supplementary figure 3*).

Since seizure activity may be dependent on the circadian rhythm, we investigated potential differences in seizure activity between the light and dark cycle in both the InfRPil-mTLE model and control animals. Interestingly, in the hippocampal CA1 recordings, all InfRPil-mTLE subgroups demonstrated significant differences between the dark and light cycles in the majority of seizure parameters analyzed (*Supplementary figure 4*, *Supplementary table 9*). For group 1P, we observed significant changes when switching between dark and light cycles regarding spike train number (*Supplementary figure 4(a)*, *Supplementary table 9*), spike train duration (*Supplementary figure 4(b)*, *Supplementary table 9*), spike train coverage (*Supplementary figure 4(c)*, *Supplementary table 9*), and single spike count (*Supplementary figure 4(g)*, *Supplementary table 9*). Similarly, group 2P displayed significant differences between dark and light cycles in all parameters, except the maximum spike train duration (*Supplementary figure 4(a–h)*, *Supplementary table 9*). Group 3P exhibited significant differences between dark and light cycles for all seizure parameters except the spike train number (*Supplementary figure 4(a)*, *Supplementary table 9*) and maximum spike train duration (*Supplementary figure 4(e)*, *Supplementary table 9*). Our results clearly demonstrate a circadian electrographic seizure pattern in the InfRPil-mTLE model, with predominant seizure activity in the dark cycle.

**Figure 4:**
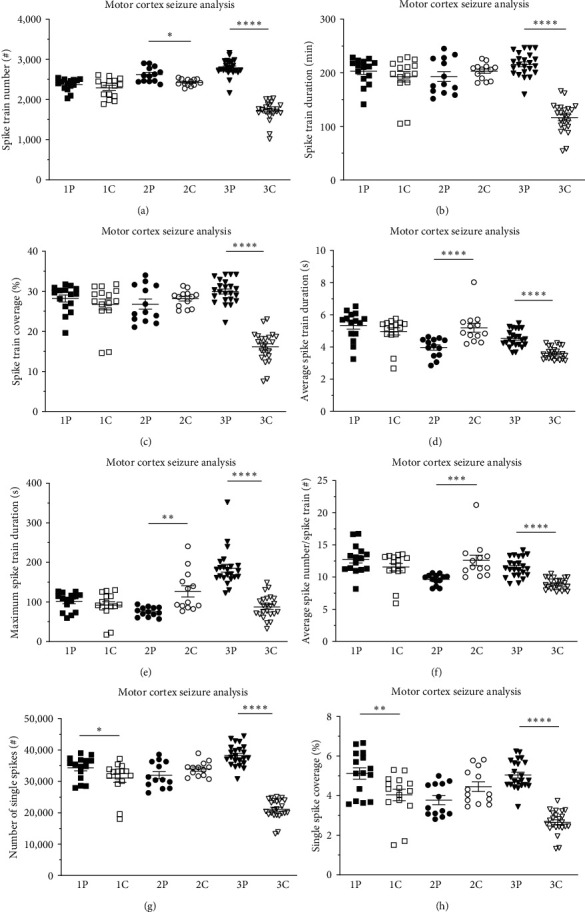
Motor cortex electroencephalographic seizure analysis of the InfRPil-mTLE model. Analysis was carried out for pilocarpine-treated rat pups (P, black symbols) which received the triphasic injection regimen at days 9, 11, and 15 pp (see also Figure 1). Sham-treated control littermates (C, open symbols) were manipulated accordingly. However, pilocarpine injection was substituted by 0.9% NaCl. The intracerebral, hippocampal EEG long-term recordings were analyzed for different seizure parameters, including (a) spike train number, (b) spike train duration (min), (c) spike train coverage (%), (d) average spike train duration (s), (e) maximum spike train duration (s), (f) average spike number per spike train, (g) total number of single spikes, and (h) single spike coverage (%). Data from experimental animals are displayed as scatter plots. Note that absolute values were averaged for a 12 hr period. In this analytical setting, potential circadian aspects are not included. For a light/dark cycle specific differentiation, see *Supplementary figure 6*. Spike activity in sham-treated rats might originate from confounding factors related to the deep electrode implantation. Results are depicted as mean ± SEM;  ^*∗*^*p* < 0.05;  ^*∗∗*^*p* < 0.01;  ^*∗∗∗*^*p* < 0.001; and  ^*∗∗∗∗*^*p* < 0.0001. A statistical overview of the hippocampal seizure parameters within the pilocarpine-treated and the sham-treated control groups is depicted in *Supplementary table 8*.

Following the hippocampal CA1 seizure analysis, we performed the same approach for the motor cortex M1 EEG recordings. Compared to the obvious, clear hippocampus seizure phenotype, we detected slightly less pronounced changes of motor cortex recordings between the InfRPil-mTLE model and sham-treated control animals, particularly for groups 1P versus 1C and 2P versus 2C (Figures 4(a), 4(b), 4(c), 4(d), 4(e), 4(f), 4(g), and 4(h)). Interestingly, there were highly significant differences in group 3P vs. 3C for all parameters. These included the spike train number (2788.7 ± 43.4 vs. 1715.1 ± 50.9, *p* < 0.0001), the total spike train duration (215.7 ± 4.5 min vs. 116.3 ± 5.8 min, *p* < 0.0001), spike train coverage (29.9% ± 0.6% vs. 16.2% ± 0.8%, *p* < 0.0001), average spike train duration (4.5 ± 0.1 s vs. 3.6 ± 0.1 s, *p* < 0.0001), maximum spike train duration (185.2 ± 9.8 s vs. 87.7 ± 6.1 s, *p* < 0.0001), average spike count/spike train (11.5 ± 0.3 vs. 8.9 ± 0.2, *p* < 0.0001), single spike count (38269.2 ± 701.4 vs. 21117.5 ± 664.1, *p* < 0.0001), and single spike coverage (5.0% ± 0.2% vs. 2.7% ± 0.1%, *p* < 0.0001). Furthermore, all three pilocarpine-treated subgroups (1P−3P) displayed an increase in almost all seizure parameters during development, underscoring once again the age-dependent, aggravating mTLE phenotype of our model (*Supplementary table 8*). In contrast, the sham-treated control group 3C exhibits a significant decrease in all seizure parameters in comparison to subgroups 1C and 2C (except for the maximum spike train duration in comparison to 1C, *Supplementary table 8*). The latter phenomenon once again indicates that ictal-like electroencephalographic activity in control animals is likely to be related to initial brain injury, which will decrease with progressive neuronal reconstitution.

In addition, relative values for the individual seizure parameters were analyzed for the cortical M1 region in the same fashion as described for the CA1 hippocampal area. The M1 motor cortex analysis of these relative seizure parameter values revealed similar results as observed for absolute values (*Supplementary figure 5*, Figure 4).

Circadian differences in seizure activity were also observed in the neocortical M1 recordings, however, to a much lesser extent (*Supplementary figure 6*, *Supplementary table 10*). This contrasts with the clear circadian pattern observed in hippocampal CA1 recordings of the InfRPil-mTLE model and control animals.

These findings further stress that pilocarpine has a stronger ictogenic effect on the hippocampus than on the motor cortex and that electrographic seizure activity predominantly originates from the hippocampal/limbic system. Interestingly, in the motor cortex, the majority of the significant alterations were detected in group 3P and 3C.

Pilocarpine-treated, as well as sham-treated control animals of group 3P and 3C, showed significantly different results between dark and light cycles for all seizure parameters, with the exception of spike train number and maximum spike train duration (*Supplementary figure 6*, *Supplementary table 10*). These findings are in line with the circadian dependency of electrographic seizure activity recorded from the CA1 region of the hippocampus (*Supplementary figure 4*, *Supplementary table 9*). Furthermore, the dark/light sensitivity seems to be age-dependent and aggravates in the older animals of both the InfRPil-mTLE and sham-treated control groups.

### 3.5. Transcriptional Alterations in the Brain of the InfRPil-mTLE Model

In total, 12 genes were analyzed in a qPCR approach to study the ictogenic effects of pilocarpine treatment on the infant rat brain (Table 4). The expression levels of voltage-gated Ca^2+^ channels (VGCCs, i.e., Ca_v_1.3, Ca_v_2.1, Ca_v_2.2, Ca_v_2.3, Ca_v_3.1, Ca_v_3.2, and Ca_v_3.3), VGCC auxiliary subunits (*β*_1–3_ and *α*_2_*δ*), and muscarinic receptors (M_1_, M_3_, and M_5_) were analyzed. These genes were studied in two different brain regions, the hippocampus and the cortex. Furthermore, animals of five different age groups were used in this experimental approach to investigate the impact of pilocarpine treatment over time (Table 1).

### 3.6. Transcriptional Alterations in the Hippocampus and Cortex

**Figure 5:**
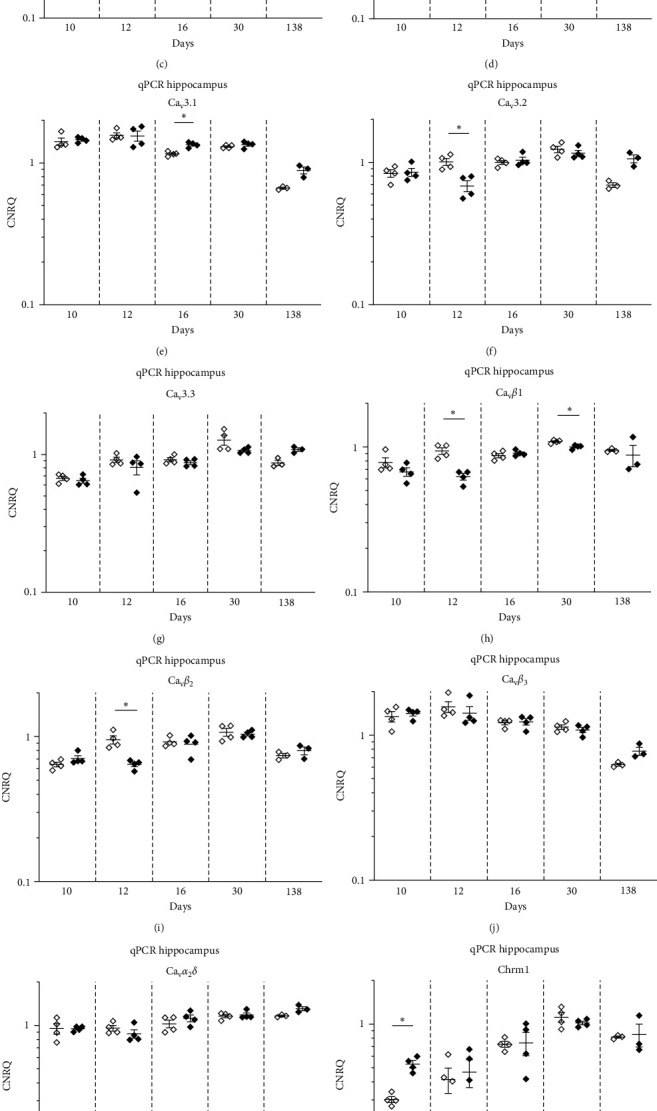
Longitudinal transcriptional alterations in the hippocampus of the InfRPil-mTLE model. Hippocampal RNA was extracted at days 10, 12, 16, 30, and 138 pp from infant/juvenile rat pups according to the triphasic regimen depicted in Figure 1. Control animals received no treatment (untreated controls) (Table 1). Note that animals used for RNA extraction were not used for radiotelemetric EEG recordings. Hippocampal RNA was screened for potential transcript alterations of different VGCC, i.e., Ca_v_1.3, Ca_v_2.1–2.3, and Ca_v_3.1–3.3 (a–g); VGCC auxiliary subunits, i.e., *β*_1–3_ and *α*_2_*δ* (h–k); and muscarinic receptors (M_1_, M_3_, and M_5_) (l–n) by qPCR. Complex and group specific longitudinal developmental changes were predominately observed in the infant mTLE rats, affecting VGCC, e.g., Ca_v_2.1 (b), Ca_v_2.3 (d), Ca_v_3.1 (e), Ca_v_3.2 (f), their auxiliary subunits *β*_1_ (h), *β*_2_ (i), and the muscarinic receptors M_1_ (l) and M_5_ (n). Most of these alterations directly correlate with the administration of pilocarpine. All transcript levels are depicted as calibrated normalized relative quantity (CNRQ). Statistical analysis (Mann–Whitney test and one-way ANOVA test) was carried out using qBase^+^ software (Biogazelle, Belgium). Results are displayed as mean ± SEM;  ^*∗*^, *p* < 0.05; T (trend), 0.5 < *p* < 0.1. For a detailed description of transcript levels, fold changes, and statistics, see Table 5. Open symbols, untreated rats; black symbols, pilocarpine-treated rats.

**Figure 6:**
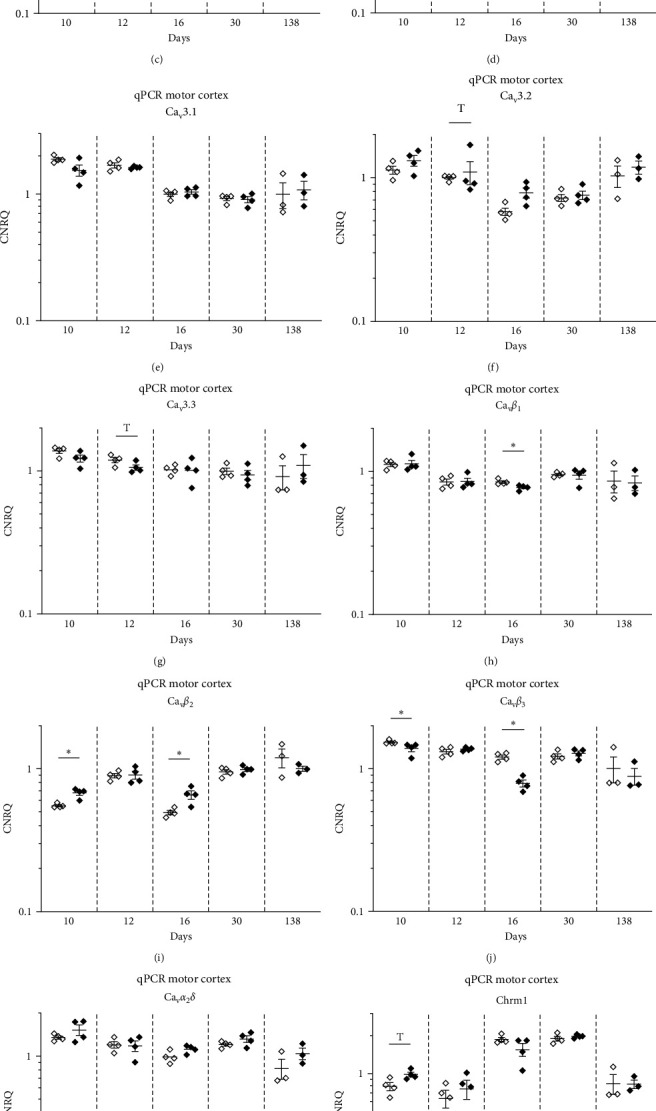
Longitudinal transcriptional alterations in the motor cortex (M1) of the InfRPil-mTLE model. Motor cortex (M1) RNA was extracted at days 10, 12, 16, 30, and 138 pp from infant/juvenile rat pups according to the triphasic regimen depicted in Figure 1. Control animals received no treatment (untreated controls) (Table 1). Note that animals used for RNA extraction were not used for radiotelemetric EEG recordings. Hippocampal RNA was screened for potential transcript alterations of different VGCC, i.e., Ca_v_1.3, Ca_v_2.1–2.3, and Ca_v_3.1–3.3 (a–g); VGCC auxiliary subunits, i.e., *β*_1–3_ and *α*_2_*δ* (h–k); and muscarinic receptors (M_1_, M_3_, and M_5_; l–n) by qPCR. Complex and group specific longitudinal developmental changes were predominately observed in the infant mTLE rats related to the Ca_v_2.1 VGCC (b), the auxiliary subunits *β*_1_ (h), *β*_2_ (i), *β*_3_ (j), and muscarinic receptors M_3_ (m) and M_5_ (n). In contrast to hippocampal transcripts (Figure 5), VGCCs were less affected in the motor cortex compared to the hippocampus in pilocarpine-treated animals. These alterations are considered to be transient phenomena during the course of epileptogenesis in the InfRPil-mTLE model. Transcript levels are depicted as calibrated normalized relative quantity (CNRQ). Statistical analysis (Mann–Whitney test and one-way ANOVA test) was carried out using qBase^+^ software (Biogazelle, Belgium). Results are consequently displayed as mean ± SEM; *p* values are defined as  ^*∗*^*p* < 0.05; T (trend), 0.05 < *p* < 0.1. For a detailed description of transcript levels, fold changes, and statistics, see Table 5. Open symbols, untreated rats; black symbols, pilocarpine-treated rats.

Voltage-gated Ca^2+^ channels (VGCCs) are categorized into high-voltage activated (HVA) and low-voltage activated (HVA) channels, depending on their activation threshold. The HVA channels can further be subdivided into the high-voltage activated (HVA) L-type Ca^2+^ channel Ca_v_1.3 and HVA non-L-type channels of the Ca_v_2 family. Neither Cav1.3 nor the *α*_2_*δ* subunit exhibited any significant changes in gene expression in pilocarpine-treated rats compared to controls at any age investigated. This held true for both the motor cortex and hippocampal tissue (Figures 5(a) and 5(k) and Figures 6(a) and 6(k)).

Interestingly, the HVA non-L-type Ca_v_2.1 Ca^2+^ channel turned out to be significantly downregulated in the hippocampus at day 12 pp (FC = −1.24, *p* < 0.05) and in the motor cortex at day 10 pp (FC = −1.25, *p* < 0.05) and at day 16 pp (FC = −1.27, *p* < 0.05). At later developmental stages however (30 and 138 days pp), no differences in Ca_v_2.1 transcript levels could be detected any more (Figures 5(b) and 6(b), Table 5).

In contrast to Ca_v_2.1, the transcript levels of Ca_v_2.2, another HVA non-L-type Ca^2+^ channel, remained unaltered in both hippocampal and motor cortex tissue. In the latter, a statistical trend for downregulation was observed at day 10 pp (FC = −1.21, 0.05 < *p* < 0.1), (Figure 5(c) and 6(c), Table 5).

The third member of the HVA non-L-type, i.e., the R-type Ca_v_2.3 Ca^2+^ channel, displayed significantly decreased transcript levels in the hippocampus (FC = −1.14, *p* < 0.05) following the second pilocarpine injection at day 12 pp. In all other experimental subgroups, Ca_v_2.3 transcript levels remained unaltered between pilocarpine-treated and untreated littermates (Figures 5(d) and 6(d), Table 5).

Next, we investigated potential alterations of the low-voltage activated (LVA) Ca^2+^ channel subfamily, which consists of three T-type Ca^2+^ channel members, i.e., Ca_v_3.1, Ca_v_3.2, and Ca_v_3.3. In the hippocampus, Ca_v_3.1 transcript levels turned out to be significantly upregulated in pilocarpine-treated animals at day 16 pp (FC = 1.16, *p* < 0.05). Interestingly, Ca_v_3.2 Ca^2+^ channels, which were reported to be involved in the etiopathogenesis of mTLE in adult mouse/rat models [32], displayed decreased transcript levels at day 12 pp in the hippocampus (FC = −1.49, *p* < 0.05) (Figures 5(e) and 5(f), Table 5). No transcriptional changes were observed for the Ca_v_3.3 T-type Ca^2+^ channel in the hippocampus for all experimental subgroups (Figure 5(g)). In the motor cortex, significant alterations in Ca_v_3 T-type Ca^2+^ channels transcription were not observed. Statistical trends were detected for Ca_v_3.2 at day 16 pp (FC = 1.35, 0.05 < *p* < 0.1) (Figure 6(f), Table 5) and for Ca_v_3.3 at day 12 pp (FC = −1.13, 0.05 < *p* < 0.1) (Figure 6(g), Table 5).

These findings clearly point to a differential, spatiotemporal role of Ca_v_3.1 and Ca_v_3.2 T-type VGCCs in the ictogenesis in the immature infant rat brain during the establishment of our InfRPil-mTLE model.

Besides the pore-forming Ca_v_-*α*_1_ subunits, various VGCC auxiliary subunits were reported to play a role in ictogenesis/epileptogenesis. The Ca_v_*β*_1_ transcript level was significantly downregulated in the pilocarpine treated group at day 12 pp (FC = −1.51, *p* < 0.05) and day 30 pp (FC = −1.09, *p* < 0.05) in the hippocampus (Figure 5(h), Table 5) and at day 16 pp (FC = −1.09, *p* < 0.05) in the cortex (Figure 6(h), Table 5).

The Ca_v_*β*_2_ subunit also exhibited reduced transcript levels in the hippocampus at day 12 pp (FC = −1.47, *p* < 0.05), but it was significantly upregulated at day 10 pp (FC = 1.23, *p* < 0.05) and at day 16 pp (FC = 1.32, *p* < 0.05) in the cortex (Figures 5(i) and 6(i), Table 5).

No alterations were observed for the Ca_v_*β*_3_ subunit in the hippocampus (Figure 5(j)). However, in the motor cortex, significantly lower transcript levels were detected in the pilocarpine treated subgroups at day 10 pp (FC = −1.11, *p* < 0.05) and at day 16 pp (FC = −1.54, *p* < 0.05) (Figure 6(j), Table 5).

As outlined above for the pore-forming, ion-conducting Ca_v_-*α*_1_ subunit, these findings also support a distinct, spatiotemporal role of *β*_1_, *β*_2_, and *β*_3_ subunits in the ictogenesis in the immature infant rat brain during the establishment of our InfRPil-mTLE model.

We next examined the transcript profiles of the muscarinic receptors Chrm1, Chrm3, and Chrm5 in the hippocampus. The Chrm1 and Chrm5 transcript levels turned out to be significantly upregulated (FC_Chrm1_ = 1.75, and FC_Chrm5_ = 1.42, *p* < 0.05). The Chrm3 receptor exhibited a statistical trend for upregulation in the pilocarpine treated group at day 10 pp (FC = 1.49, 0.5 < *p* < 0.1) (Figures 5(l), 5(m), and 5(n), Table 5).

In the cortex, significantly increased transcription was detected at day 12 pp for the muscarinic receptor Chrm3 (FC = 1.17, *p* < 0.05) and at day 16 pp for Chrm5 (FC = 3.07, *p* < 0.05) (Figure 6(m) and 6(n), Table 5). In contrast, the Chrm1 receptor displayed a statistical trend for upregulation at day 10 pp (FC = 1.25, 0.5 < *p* < 0.1) (Figure 6(l), Table 5).

Interestingly, when the permanent effects of pilocarpine were investigated on day 138 pp (qPCR group 138d), the expression levels of the receptors tested did not significantly vary between pilocarpine-treated and pilocarpine-untreated animals. Although the FC values for the 138d group were in the range of 1.25–1.53, a range that resulted in significant differences between pilocarpine-treated and pilocarpine-untreated younger groups, group 138d did not reach significant transcription level changes in any gene tested. One reason might be that group 138d included a smaller number of animals in comparison to the rest of the RT-qPCR groups (three animals instead of four) (Tables 1 and 5).

## 4. Discussion

### 4.1. The Novel Electroencephalographic Infant Rat Pilocarpine Model of mTLE (EEG-InfRPilo-mTLE): Strain-, Sex-, and Age-Specific Aspects

In this manuscript, we present and characterize a novel rat pilocarpine model of mTLE established in infant animals. It exhibits spontaneous and recurrent electroencephalographic hippocampal seizures in adulthood and a survival rate of 80.3%. We observed that administration of three consecutive pilocarpine injections in rats of 9, 11, and 15 days of age generates a stable electrographic phenotype that can be detected in both the hippocampus and the motor cortex in the mature brain. In previous studies [16, 17, 18, 19, 20, 21, 59], various pilocarpine concentrations were administrated (170–350 mg/kg) in neonatal, infant, and juvenile rats (aged 7–21 days) using various injection schemes. Although the animals exhibited behavioral impairment and histological changes, the mTLE models with spontaneous epileptic activity needed improvement [16, 17, 18, 19, 20, 21, 59].

In general, rats represent the most frequently used species in mTLE modeling. Most studies on rat mTLE have been carried out in Wistar rats and focused on males in order to restrict the potential implications of the estrous cycle, sex hormones, and their effects on seizure activity [60, 61]. As regards mTLE models, no differences were described in behavior, EEG, and SRS in male and female Wistar rats, although this aspect was not explicitly investigated [16, 62, 63]. As estrous cycle-dependent fluctuations in steroids were reported to severely influence pilocarpine related survival and latencies of seizure onset and considering the protective role of female sex hormones against pilocarpine induced SE [64], we decided to use male rats in our study.

### 4.2. Experimental Animal Pretreatment in EEG-InfRPilo-mTLE

Anticholinergic drugs that do not cross the BBB are widely used to counteract peripheral pilocarpine-induced cholinergic stimulation, e.g., bradycardia, piloerection, salivation, tremor, chromodacryorrhea, and diarrhea [65]. At low doses (1 mg/kg), alpha-methylscopolamine does not inhibit pilocarpine effects in the CNS. However, higher doses (i.e., 20 mg/kg, s.c.) were shown to prevent induction of SE in rats [3, 4, 66]. In our EEG-InfRPilo-mTLE model, we followed the established approach of low-dose alpha-methylscopolamine administration (1 mg/kg, s.c.) 30 min prior the triphasic pilocarpine injection regimen. The experimental animals of days 9, 11, and 15 pp well tolerated this muscarinic antagonist.

### 4.3. Pilocarpine Route of Administration in EEG-InfRPilo-mTLE

Previous studies elucidated that the effectiveness of intraperitoneal and direct intrahippocampal injections of pilocarpine turned out to be comparable in terms of behavioral, electrographic, and neuropathological alterations, and the induction of SE and SRS [1]. Despite this potential superiority of direct intrahippocampal pilocarpine administration in terms of lethality and seizure characteristics, we decided to use systemic pilocarpine injection, as we wanted to minimize potential early hippocampal damage due to the injection, and potential interference and/or impairment of subsequent telemetric EEG electrode implantations and recordings [1, 67].

### 4.4. Pilocarpine Dose Finding and Triphasic (Fractional) Application in the EEG-InfRPilo-mTLE Model

Previous findings demonstrated that pilocarpine effects in mTLE generation are dose-dependent. Many studies compared pilocarpine doses of 100–400 mg/kg in adult male Wistar rats [3, 9]. Dose independently, rats first became immobile, followed by gustatory and olfactory automatisms, e.g., salivation, orofacial movements, and vibrissae twitching. With the highest dose of pilocarpine, i.e., 400 mg/kg, animals also displayed limbic motor seizures [3, 9, 16]. Thus, with increasing the pilocarpine doses, the likelihood of rats exhibiting the complete syndrome, and reduced latency to SE/SRS, is also increased. Unfortunately, the lethality also increased [9, 65].

As a consequence, multiple low-dose protocols (using, e.g., 5 mg/kg KA i.p. every hour) were developed, based on the individual responses of the experimental animals [68]. Previously, researchers already tried to reduce high mortality by repetitive pilocarpine injections, e.g., with an initial dosage of 200 mg/kg, followed by additional dosages of 100 mg/kg until SE finally evolved [69]. Unfortunately, this approach was described to be associated with a delay of SE onset (up to 6 hr) [69]. The variability in mortality and fraction of rats that develop SE and SRS/SRES can be related to strain, sex, and age differences. For example, aged rats (24 months old) are less susceptible to KA-induced excitotoxicity than 3−20-month-old rats [70]. Juvenile rats (35–40 days old) turned out to be more susceptible and displayed more consistent motor seizures post-KA treatment than adult rats (70–90 days old) [71]. Notably, young rats (up to 25 days old) treated with pilocarpine (or KA) to induce SE do not exhibit severe neuronal cell loss and network restructuring. Indeed, in young rats, hippocampal neurons were more resistant and exhibited increased survival and synaptic reorganization which made it more difficult for SRS to emerge [20, 72]. This increased resistance of infant rats explains the necessity of the repetitive, low-dose pilocarpine administration in our EEG-InfRPilo-mTLE model. Our preliminary studies suggested that a triphasic injection regimen with pilocarpine (180 mg/kg i.p.) at days 9, 11, and 15 pp was most suitable for our purposes with an overall survival rate of 80.3%.

### 4.5. Duration of SE in the EEG-InfRPilo-mTLE Model

It is well-known that the duration of SE is critical to the development of SRSs and morphological brain damage. However, the interdependence between both factors turned out to be complex in different settings. Some authors found that reducing SE duration (1–2 hr) increased the latency to first occurrence of SRS and reduced the seizure frequency as well as the occurrence of severe neuropathological alterations in adult male Wistar rats (treated with 300–320 mg/kg pilocarpine, 10 mg/kg diazepam, and 30 mg/kg pentobarbital) [51]. When SE is terminated prematurely, neuronal loss is limited and synaptic reorganization and epilepsy fail to emerge [51, 73, 74, 75]. The SE duration of 30 min was proposed as a minimal duration to develop subsequent SRS [51]. Curia et al. [1] and Klitgaard et al. [76], for example, induced SRS in rats that had exhibited 30 min SE with a latent period of 3 weeks. Clearly, the neuropathological alterations increased with prolonged SE duration [9, 51]. Importantly, the route of administration of benzodiazepines and/or barbiturates is likely to influence this phenomenon [6, 51, 76, 77]. During the course of our preliminary studies, it turned out that infant rats do not tolerate acute injection of higher pilocarpine doses that are normally administered in adults. Lower pilocarpine doses where thus associated with shorter SE. As prolonged SE also coincided with increased lethality, we decided to terminate SE following a 45 min period.

### 4.6. Termination of SE in the EEG-InfRPilo-mTLE Model

Benzodiazepines, such as diazepam (5–10 mg/kg), are known to impair/prevent the development of pilocarpine induced motor and EEG alterations as well as the subsequent morphological changes in rats [3, 78]. Clearly, single diazepam injection (20 mg/kg, i.p.) 30, 60, 120, or 180 min after the SE onset significantly influenced lethality and SRSs depending on SE duration. Without diazepam, SE was reported to remit spontaneously within few hours after pilocarpine treatment [51]. In infant rats, the combined administration of benzodiazepines and barbiturates or antiepileptic drugs to terminate SE did not seem feasible for us, as infants are highly sensitive to polypharmaceutical approaches. We applied a restrictive, monotherapeutic regimen using repetitive, short-interval low-dose diazepam injections (0.0375–0.1 mg i.p.) until cessation of SE. Single, high-dose diazepam injections turned out be lethal in our infant rats.

It has been demonstrated that following termination of SE by diazepam, pentobarbital, or phenytoin [50, 51, 74], the latent period duration changes significantly as a function of the time intervals of continuous seizure activity in the acute seizure period. Rats that perceived a 30 min SE and which were then treated with a single (i.p.) injection of diazepam (10 mg/kg) and pentobarbital (30 mg/kg) did not develop SRSs [51]. Rats that perceived SE of 1, 2, 6, or more hours exhibited latent periods of 52, 38, 17, and 14 days, respectively. Contrary to this, Biagini et al. [50] found that the latent period is progressively shortened by decreasing the SE length. Notably, recent studies based on video-EEG recordings revealed an average latent period of 7 days after a 2 hr SE [6]. Lemos and Cavalheiro [51] used Wistar rats weighing 200–250 g [51], whereas Goffin et al. [6] analyzed Wistar rats with a body weight range of 285–350 g that was similar to that of the Sprague-Dawley rats (270–300 g) used by Biagini et al. [50]. Obviously, strain and age are not likely to be responsible for these discrepancies. After all, the pharmacological approach in SE termination has a major impact: rats developing SRSs more rapidly were treated only with diazepam (20 mg/kg, i.p.) [6, 50]. The monopharmaceutical approach of SE termination using diazepam thus turned out to be the most suitable regimen in our EEG-InfRPilo-mTLE model.

### 4.7. The Latent Period in the EEG-InfRPilo-mTLE Model

The SE and its termination using diazepam is followed by a latent period that precedes the occurrence of SRS. This latent period was reported to be accompanied by impairment of long-term plasticity in the hippocampus in young rats [79]. Apart from rare events in which rats were reported to present continuous seizures in the first 3 days after SE [10], pilocarpine-treated rats generally present normal behavior and EEG activity during the latent period [1]. For the latent period in our model, we can confirm that rats did not exhibit abnormal behavioral patterns. For experimental reasons, radiotelemetric EEG electrodes and transmitters were first implanted in group I aged 36–38 days, i.e., after weaning with an almost fully developed skull and brain. As our model turned out to show electroencephalographic seizures without obvious motor seizures, we cannot comment on a potential latent period and save the fact that the latent period lasts less than 35 days, because EEG recordings from group I (days 43–50 pp) display SRES.

### 4.8. EEG Alterations following Initial Pilocarpine Treatment

EEG alterations upon initial pilocarpine injections show two characteristic dose-dependent stages: first, low-voltage fast activity apparent in the cortex and amygdala, accompanied by theta rhythm in the hippocampus, and next, hippocampal theta replaced by high-voltage fast activity with prominent isolated high-voltage spikes [1]. The SE is characterized by ictal and interictal discharges in the EEG, and these alterations are often also associated with behavioral changes. In rats treated with low doses of pilocarpine (100–200 mg/kg), this EEG pattern can last up to 2 hr, and the normal EEG profile recovers shortly after, if seizure activity is terminated before. The highest pilocarpine doses, e.g., 400 mg/kg, induced electroencephalographic seizures that were preceded by high-voltage fast activity and spiking and followed by irregular postictal depression periods. Note that we did not record EEGs following the initial pilocarpine treatments in our InfRPilo-mTLE model, as EEG electrode and transmitter implantation was carried out at later developmental stages.

### 4.9. Characteristics of SRES in the EEG-InfRPilo-mTLE Model

The chronic period post-epileptogenesis is characterized by SRSs. Similar to the silent/latent period, the chronic phase can be heterogenous depending on the individual protocol design of the pilocarpine mTLE model. At the behavioral level, SRSs were classified by Pitkänen [80] based on the following characteristics: (1) staring and mouth clonus; (2) automatisms; (3) monolateral forelimb clonus; (4) bilateral forelimb clonus; (5) bilateral forelimb clonus with rearing and falling; and (6) tonic–clonic seizures. This classification was further simplified by Goffin et al. [6] who summarized stages 1–3 as partial seizures and stages 4–6 as secondarily generalized seizures. The SRSs start approximately 7 days after SE as partial seizures and subsequently evolved into generalized seizures [6]. Interestingly, shorter silent periods (3–5 days) were associated with high number of SRSs, whereas longer latent periods (28–30 days) were associated with a reduced number of SRSs during 135 days of observation [1, 81]. It has been reported that SRSs recur in clusters, peaking every 5–8 days [6] or more [81]. Besides, SRSs occur relatively regular postestablishment of mTLE [20]. Notably, pilocarpine-treated rats without SE can also develop SRS after a prolonged latent period. Thus, low-dose pilocarpine dosage(s) and indifferent SE activity is clearly no knock-out criterion for establishment of SRS or SRES [14].

Of note, the seizure frequencies were found to vary based on the technique used to monitor the rats (visual inspection, video monitoring, or continuous video-EEG recording) and the type of seizures scored (partial or generalized). Apparent motor seizure activity was not detected in our EEG-InfRPilo-mTLE model. As outlined in the *Results* section, our model turned out to exhibit SRES that were characterized by high-amplitude spikes and/or spike trains and bursts of spike activity that often originated from the hippocampus and spread to the cortex, as has been reported previously [10]. The SRES in our model rarely last more than 60 s and are often followed by depressed EEG background activity and frequent interictal spikes. From our long-term EEG recordings from both the cortex and hippocampus, we can conclude that seizure activity predominantly originated in the hippocampus and spread to the cortex. This observation is in line with reports by Toyoda et al. [82] who claimed that the ventral hippocampus and subiculum are dominant onset sites and not the neocortex. In rare cases, however, we detected isolated seizure activity in the neocortex, which is in contrast to reports by Calvalheiro et al. [10]. Notably, the overall seizure severity increased with time (groups 1P to 3P covering a total duration of 99 days; Figures 3 and 4; *Supplementary tables 7* and *8*). These findings stress that the SRSs in our EEG-InfRPilo-mTLE model do not decrease over time and that we have no indication of potential counteracting, neuro-rescue, and antiepileptogenic processes following the establishment of our model.

It was previously reported that seizure frequency was higher during the diurnal period (i.e., from 7:00 a.m. to 7:00 p.m.) when the animals were kept in a 12 hr dark/light cycle [81]. Similarly, video-EEG recordings suggested that about 67% of daily seizures occur during the light phase [6]. Furthermore, interictal activity was reported to be more intense during the seizure-free periods and during sleep and not detectable during motor behavior and REM sleep [81]. These previous observations are in contrast to the seizure architecture in our EEG-InfRPilo-mTLE model, in which seizure activity is significantly enhanced during the dark period compared to the light cycle.

### 4.10. Developmental and Morphological Alterations in EEG-InfRPilo-mTLE

Similar to the EEG, neuropathological alterations in pilocarpine treated animals were shown to be dose-dependent. Even lowest doses of pilocarpine, e.g., 100 mg/kg, were capable of damaging the piriform cortex and anterior olfactory nuclei, extending then to the cortex, amygdala, and basal nuclei. With higher doses, e.g., 200 mg/kg, rats also exhibited severe limbic motor seizures and damage in the medio-dorsal thalamic nuclei and neocortex [1, 4]. In our approach, we did not carry out morphological studies, a task which has to be addressed in the future. However, developmental alterations during the course of establishment of the mTLE model are also reflected by body weight. The latter is known to decrease following SE (10%–20%) but was reported to regain pretreatment levels soon after the procedure, e.g., within 1 week [11]. We observed a similar phenomenon during the course of our triphasic injection regimen. One day after each pilocarpine injection, body weight was significantly reduced compared to sham-treated controls. However, this developmental retardation was successfully compensated in the treated group shortly after, and no significant impairment in body weight was detected at later stages.

### 4.11. Transcriptional Alterations in the EEG-InfRPilo-mTLE Model

In addition to the EEG evaluation, we also performed RT-qPCR of selected genes from hippocampal and motor cortex tissue of our EEG-InfRPilo-mTLE model. We characterized some factors that might underlie the dynamic evolution of functional abnormalities after early SE that may enhance our understanding of the fundamental pathophysiology and related consequences of recurrent SE in the immature human CNS. Although there are several reports focusing on the histological, morphological, and behavioral evaluation of pilocarpine-treated neonatal/infant/juvenile rats [26, 55, 57, 83], there is limited research on transcript analysis of acute repetitive pilocarpine injection as well as in the long-term range. According to previous reports, histological alterations and neuronal loss can occur early within 24–72 hr in the hippocampal CA1 region of the neonatal/infant/juvenile rat brain, after pilocarpine treatment [55, 57, 83, 84]. Although histological alterations were compensated 2 months following pilocarpine injection, cognitive impairment as well as hippocampal volume reduction was still observed in adult animals that were pilocarpine-treated between 1–15 days of age [17, 19, 26]. Considering the cellular and behavioral changes in pilocarpine-treated neonatal and juvenile rats, we aimed to investigate the short- as well as the long-term transcriptional alterations of selected gene candidates known to be important in epileptogenesis. As the role of Ca^2+^ channels and their auxiliary subunits in epileptogenesis has previous been suggested [32, 85, 86], we further investigated the transcript levels of different VGCCs (i.e., Ca_v_1.3, Ca_v_2.1–2.3, and Ca_v_3.1–3.3) and VGCC auxiliary subunits (*β*_1–3_ and *α*_2_*δ*_1–4_). Furthermore, we searched for potential transcriptional alterations of muscarinic acetylcholine receptors (M_1_, M_3_, and M_5_). Complex alterations were observed over the course of the triphasic injection regimen representing the process of initial pharmacological brain damage/kindling and epileptogenesis from the infant to the mature brain. During this process, modifications in Ca_v_2.1, Ca_v_2.3, Ca_v_3.1, and Ca_v_3.2 transcript levels manifested in the hippocampus, predominantly following the second pilocarpine injection regimen, stressing that brain injury mediated epileptogenesis is not a monofactorial but rather a multifactorial process. Interestingly, following the second pilocarpine injection, Ca_v_2.1, Ca_v_2.3, and Ca_v_3.2 genes and also the auxiliary subunits *β*_1_ and *β*_2_ were downregulated with the exception of Ca_v_3.1 which was upregulated. Previously, Becker et al. [32] and other groups found that Ca_v_3.2 T-type VGCCs were upregulated in the hippocampus in the adult mouse mTLE model, likely serving as key player in mTLE epileptogenesis [32, 87]. The Becker group [32] used both Ca_v_3.2^+/+^ and Ca_v_3.2^−/−^ male mice (60 days old) [88] and Wistar rats (150–200 g), and the animals received only a single pilocarpine dose of 330 mg/kg, s.c.. Importantly, in the same study [32], a transient increase in transcript levels of *β*_1_ and *β*_2_ was also reported, whereas *β*_3_ was reduced at later stages. Given the potential epileptogenicity of the aforementioned channels and auxiliary subunits, one might speculate that the hippocampus of our infant rat model reactively downregulates the related transcripts as an adaptive reaction to the pharmacological induction of hyperexcitability, excitotoxicity, and brain injury. Transcript levels of the *α*_2_*δ* auxiliary subunits remained unchanged in our study similar to the report of Becker et al. [32].

In addition, distinct differences in transcription pattern were observed in the cortex of our EEG-InfRPil-mTLE model. Ca_v_2.1 exhibited significant reduction following the first and third pilocarpine injection. Furthermore, a significant reduction of *β*_1_ (day 16) and *β*_3_ (days 10 and 16) and a significant increase in *β*_2_ (days 10 and 16) transcript levels were observed in the cortex of our EEG-InfRPil-mTLE model. Finally, in both the hippocampal and cortical probes, a significant increase in M_1_, M_3_, and M_5_ transcript levels was detected compared to untreated animals.

In the adult model of pilocarpine mTLE induction, a transient hippocampal transcriptional alteration was reported for Ca_v_3.2 but not for Ca_v_1.1, Ca_v_1.2, and Ca_v_2.1–2.3. This alteration appeared 2–3 days post-SE but disappeared 10 days post-SE [32]. We were not able to confirm these findings in our EEG-InfRPil-mTLE model. RT-qPCR results showed high FC in genes on day 138 pp like Ca_v_2.3, Ca_v_3.1–3.3, auxiliary subunit *β*_3_, and M_5_ for the hippocampus and Ca_v_1.3, Ca_v_2.2, Ca_v_2.3, and *α*_2_*δ* and M_5_ for the cortex (Table 5). Although these alterations did not reach significance due to the small sample size (group 138 days, *n* = 3; Table 4), the high FC in the adult age indicates permanent hippocampal and cortical molecular changes which could explain cognitive impairments [17, 19] and hippocampal volume reduction [26] as reported previously. These findings again indicate that infant mTLE is not based on a monofactorial event, but it is rather mediated by the complex spatial and temporal expression patterns of multiple genes and their physiological interdependence. Whereas in the adult rat model of mTLE a single episode of SE is sufficient to develop molecular changes within the brain triggering epileptogenesis and changes in neuron membrane properties and synaptic function [32, 89], this does not turn out to be effective in the infant rats.

Considering the cellular and behavioral changes in pilocarpine-treated infant rats reported in other studies [26, 55, 57, 72, 83, 90], our RT-qPCR results suggest that the SRES in our rat mTLE model are the result of a complex molecular and neuronal dysregulation, originating from the triphasic injection regimen in the infant brains.

Finally, one should emphasize that other molecular studies have been carried out in the past, using different species, strains, or ages in mTLE models. Alterations of VGCCs in the mouse brain following SE included significant expression changes of Ca_v_1.2, Ca_v_1.3, and Ca_v_2.1 in different hippocampal neuronal ensembles [91]. The complex up- or downregulation of HVA Ca_v_1.2, Ca_v_1.3, or Ca_v_2.1 channels in different interneuron entities may thus be related to changes of their plasticity [91]. Enhanced *β*_1_ and *β*_2_ immunoreactivity in neuronal cell bodies and a shift of *β*_1_ and *β*_2_ auxiliary subunits were described in dendrites of highly sclerotic hippocampal areas characteristic of Ammon's horn sclerosis. The increased Ca^2+^ currents related to altered *β* expression is likely to contribute to enhanced synaptic excitability [92]. Dysregulation of VGCC thus seems to be a consistent trans-species feature in mTLE models. Finally, there have recently been attempts to characterize the entire postpilocarpine transcriptome in mTLE models [86, 93, 94].

### 4.12. Study Limitations

Young rats exhibit increased susceptibility to seizure induction and therefore require lower pilocarpine doses [80, 95, 96]. The manifestation of seizures becomes more obvious with increased age [95, 96]. Later on in adulthood, these animals only display minimal or undetectable cellular damage [97]. In the acute and short-term phase, however, SE can induce neuronal injury in the amygdala, hippocampus, and mediodorsal thalamic nucleus [56, 90], but young rats do not show prominent reorganization that is characteristic for adult rats [96, 97].

SE animal models have intrinsic limitations, e.g., the pattern of neuronal cell loss differs from that seen in patients. In the latter, cell loss is less symmetric [98]. In addition, rodent SE models also display more neuronal degradation in extrahippocampal regions [2], e.g., in the olfactory cortex [2]. Importantly, reduction of CA1 pyramidal cells is not consistently observed in rodent models. Notably, animal models normally exhibit shorter latent periods and advanced age at which the initial brain injury takes place compared to patients. This aspect stresses the importance of neonatal/juvenile rat models of mTLE in translational categories.

The fast development of SE following pilocarpine injections at days 9, 11, and 15 pp in our new model is in line with previous findings on the susceptibility of the immature CNS to epileptogenesis [99, 100]. Although the immature brain is seizure-prone, it is known to be less vulnerable to seizure-triggered pharmacological brain injury and subsequent synaptic reorganization [101, 102]. Consistently, no evident neuronal loss was observed in adult rats subjected to multiple SE in early life when studied with a standard cresyl violet staining approach [103]. However, the TUNEL method revealed increased apoptotic neuronal death and long-lasting maladaptive changes in several brain areas in adult rats that had undergone multiple SE during early infancy (P7–P9). Although there is no evidence for late neuronal injury (cell loss) or degeneration in this model, these animals did exhibit enduring and latent epileptogenesis [17, 18, 103, 104].

Multiple pilocarpine-induced SE at days 9, 11, and 15 pp can thus induce devastating neuronal alterations that are revealed by complex qualitative and quantitative EEG alterations and that can be detected for several weeks following the initial triggering insult. The early recurrent seizures may thus lead to a chronic epileptogenic environment that progressively escalates into adulthood. As has been elaborated in the early description of the pilocarpine model [10], electroencephalographic, subconvulsive epileptogenesis can serve as endogenous kindling process on CNS hyperexcitability. As a matter of fact, seizures during early development were identified as a central cause for mature brain dysfunction [105]. It is speculated that neuronal networks of the immature brain may cope with acute injury, e.g., monophasic pilocarpine administration, before day 10 pp without exhibiting post hoc functional and structural abnormalities. Multiple SE episodes, however, obviously exceed this regenerative capacity of the neonatal/juvenile brain finally resulting in neuronal circuit dysfunction and persistent hyperexcitability. This aspects requires further investigations in the future.

## 5. Conclusions

Several adult rat models of mTLE have been developed in the past. However, infant rat models of mTLE have not been described yet. Here, we presented the InfRPil-mTLE approach, a novel infant rat model of mTLE which is based on a triphasic injection regimen. The latter consists of low-dose pilocarpine administrations (180 mg/kg. ip.) on days 9, 11, and 15 pp, a survival rate of >80%, and typical SRES in both the hippocampus and cortex that persist into adulthood. In contrast to previous studies [16, 17, 18, 19, 20, 52, 59], all surviving pups of our model exhibit chronic electroencephalographic seizure activity/SERS in the hippocampus and cortex as early as 4 weeks following the final pilocarpine injection regimen at day 15 pp. These findings demonstrate that our model is more efficient in terms of chronic electroencephalographic seizure activity than any other pilocarpine induced model of mTLE described in literature [13]. In addition, RT-qPCR analysis of selected gene candidates revealed the downregulation of Ca_v_2.1, Ca_v_2.3, Ca_v_3.2, *β*_1_, *β*_2_, M_1_, and M_3_ in the hippocampus and cortex of our InfRPil mTLE model. From a translational point of view, our model could serve as a blueprint for childhood epileptic disorders and further enhance antiepileptic drug research and development in the future.

## Figures and Tables

**Table 1:** Experimental InfRPil-mTLE animals and grouping for RT-qPCR studies.

Experimental parameters	RT-qPCR groups									
	Group 10d		Group 12d		Group 16d		Group 30d		Group 138d	
	Treated	Untreated	Treated	Untreated	Treated	Untreated	Treated	Untreated	Treated	Untreated
Group size (#), all ♂	4	4	4	4	4	4	4	4	3	3
Age (days pp)	10	10	12	12	16	16	30	30	138	138
Pilocarpine injections (#)	One injection: day 9 pp	0	Two injections: days 9 and 11 pp	0	Three injections: days 9, 11, and 15 pp	0	Three injections: days 9, 11, and 15 pp	0	Three injections: 9, 11, 15 days pp	0
Body weight (g)	Day 9 pp: 18.6 ± 0.4	—	Day 11 pp: 23.2 ± 2.1	—	Day 15 pp: 36.1 ± 1.9	—	—	—	—	—
	Day 10 pp: 17.3 ± 0.4 (↓, *p* < 0.0001, compared to day 10 pp untreated animals)	Day 10 pp: 25.2 ± 0.8	Day 12 pp: 24.5 ± 2.0 (↓, *p*=0.016, compared to day 12pp untreated animals)	Day 12 pp: 31.4 ± 0.6	Day 16 pp: 36.9 ± 1.7 (↓, *p*=0.001, compared to day 16 pp untreated animals)	Day 16 pp: 44.8 ± 1.2	Day 30 pp: 104.2 ± 6.4 (*p*=0.559, compared to day 30 pp untreated animals)	Day 30 pp: 109.5 ± 5.9	Day 138 pp: 588.6 ± 9.8 (*p*=0.076, compared to day 138day pp untreated animals)	Day 138 pp: 560.6 ± 6.6

Rats investigated here for developmental/body weight analysis were also used for qPCR studies and not for EEG radiotelemetry to avoid potential interference with the radiofrequency transmitter implantation procedure. As outlined in Figure 1, rats were sacrificed 1 day after the first, second, and third triphasic injection regime, corresponding to days 10, 12, and 16 pp. In addition, brains of treated and untreated rats were extirpated 2 days after weaning (day 30 pp) and at a later stage (138 days pp). Statistical comparison of body weight between pilocarpine-treated and pilocarpine-untreated animals revealed statistically significant decrease of body weight in pilocarpine-treated animals in comparison to untreated controls (at days 10, 12, and 16 pp), indicating a developmental impairment upon seizure induction. At later stages (days 30 and 138 pp), these developmental discrepancies are abolished.

**Table 2:** Experimental animals and grouping for radiotelemetry-based electroencephalographic seizure analysis.

Groups	Transmitter implantation		Recording age (days)	# of animals/group; all ♂
	Age (days)	Body weight (g)		
1P	36.8 ± 0.3	169.8 ± 7.2	43–50	9
1C	37.7 ± 0.1	196.3 ± 5.1 (1C vs. 1 P, *p*=0.0059)	43–50	12
2P	58.4 ± 0.2	307.9 ± 4.2	64–70	9
2C	57.9 ± 0.2	348.8 ± 11.3 (2C vs. 2 P, *p*=0.0059)	64–70	11
3P	122.9 ± 0.7	527.0 ± 11.4	127–134 and 137–142	24
3C	119.9 ± 0.3	542.2 ± 12.9 (3C vs. 3 P, *p*=0.4079, ns)	127–134 and 137–142	13

All pilocarpine-treated animals were administered a triphasic regimen at days 9, 11, and 15 pp as outlined in Figure 1. Sham-treated control groups followed the same injection regimen but received 0.9% NaCl instead of pilocarpine. Experimental animals were again weighed prior to radiotransmitter implantation. It turned out that in the pilocarpine-treated study groups 1P and 2P, body weight was significantly reduced compared to sham-treated rats at the time point of implantation. This developmental impairment upon pilocarpine treatment was abolished at a later stage (see 3P and 3C), paralleling the observations described in Table 1 for RT-qPCR groups.

**Table 3:** Weight- and age-related scaling of stereotactic coordinates for electrode positioning in infant rats.

Region	Coordinates	Animal weight	
		<250 g (80%) Group 1P/1C	>300 g (100%) Groups 2P/2C and 3P/3C
Hippocampus (CA1)	Anterior–posterior (bregma)	−3.0 mm	−3.8 mm
	Lateral (paramedian sagittal)	+2.0 mm	+2.5 mm
	Dorsal–ventral (depth)	2.1 mm	2.6 mm

Motor Cortex (M1)	Anterior–posterior (bregma)	2.0 mm	2.5 mm
	Lateral (paramedian sagittal)	−2.4 mm	−3.0 mm
	Dorsal–ventral (depth)	0 mm	0 mm

In literature, rat brain stereotaxic coordinates are derived from adult male Wistar rats with body weights ranging from 270 to 310 g and claiming validity within the range of 250–350 g [38, 39, 40]. In our study, stereotaxic coordinates had to be adapted by 80% of the reference values for telemetry groups 1P and 1C since the animals in those groups were still adolescent with lower body weight (169.8 ± 7.2 g and 196.3 ± 5.1 g, respectively; see also Table 2). For further experimental subgroup characteristics, please refer to Table 2.

**Table 4:** Genes and related primer pair sequences used for the RT-qPCR experiments.

Protein/gene	Forward sequence (5′–3′)	Reverse sequence (5′–3′)	Size (bp)
Ca_v_1.3/cacna1d^1^	CCCAATGGAGGCATCACTAACTTTG	CACGGCAGCCCTGCACCTCCTGC	604
Ca_v_2.1/cacna1a^2^	GTCGTGGTGCTAACAGGCATC	ACGAACCGCCCTCAGTGTC	72
Ca_v_2.2/cacna1b^2^	TCATCGGCCTCGAGTTCTATATG	TCACCCACAGGCTCTGCAT	79
Ca_v_2.3/cacna1e^2^	TGAGGTCGTTTGGGCAATCT	TCCTTAGGAGCCGGAGAGCT	77
Ca_v_3.1/cacna1g^2^	ACCCTGGCAAGCTTCTCTGA	TTTCGGAGGATGTACACCAGGT	74
Ca_v_3.2/cacna1h^2^	ATGTCATCACCATGTCCATGGA	ACGTAGTTGCAGTACTTGAGGGCC	76
Ca_v_3.3/cacna1i^2^	TCATCCGTATCATGCGTGTTC	GGCCCGCATTCCTGTG	74
Ca_v_ß1/cacnb1^2^	GGCTGTGAGGTTGGTTTCATC	TGGCGCAGCGTCTGTTC	77
Ca_v_ß2/cacnb2^2^	CCCCAGTAAGCACGCAATAATAG	AAAATCCTTTCAATTTCACTCTGAACTT	81
Ca_v_ß3/cacnb3^2^	CAGATGCCTACCAGGACCTGTAC	TGCCCGTTAGCACTGGGT	70
Ca_v_*α*_2_*δ*_1−4_/cacna2d^2^	AATGCTCAGGATGTGAGTTGTTTC	TTATTCACCGCATCCTTCAGC	83
M_1_/Chrm1^3^	GCACAGGCACCCACCAAGCAG	AGAGCAGCAGCAGGCGGAACG	373
M_3_/Chrm3^3^	GTCTGGCTTGGGTCATCTCCT	GCTGCTGCTGTGGTCTTGGTC	434
M_5_/Chrm5^4^	TGGTCATCCTCCCGTAGAAGCA	GCTACAGTTGGTAACCTGCTCAG	102
HPRT/hprt^5^	GACTTTGCTTTCCTTGGTCA	AGTCAAGGGCATATCCAACA	152

^1^Weiergräber et al. [37]; ^2^Becker et al. [32] and personal communication; ^3^Wei et al. [45]; ^4^OriGene Technologies GmbH, Germany; ^5^housekeeping gene for normalization and quantification of the qPCR results (Biomol GmbH, Germany). RNA was isolated from the motor cortex (M1) and hippocampus of pilocarpine-treated and untreated rats as outlined in Figure 1 (see also Table 1). Note that tissue extirpation was carried out 1 day after each triphasic injection regimen (days 9, 11, and 15 pp) and at later stages, i.e., days 30 and 138 pp. Transcripts of VGCC *α*_1_-subunits (Ca_v_1.3, Ca_v_2.1–2.3, and Ca_v_3.1–3.3), VGCC auxiliary subunits (*β*_1–3_ and *α*_2_*δ*_1–4_), and muscarinic receptors (M_1_, M_3_, and M_5_) were investigated. The forward and reverse primer sequences (5′−3′) as well as the expected sizes of the amplicons are given for the individual protein/gene candidates.

**Table 5:** Transcript fold changes (FC) of hippocampal and motor cortex (M1) gene candidates from the novel infant rat model of mTLE.

(A)	Hippocampus (FC)				
	qPCR Groups				
Protein/gene	10 days	12 days	16 days	30 days	138 days
Ca_v_1.3/cacna1d	1.22	−1.02	−1.06	−1.07	1.08
Ca_v_2.1/cacna1a	−1.15	−1.24	−1.01	−1.05	1.20
Ca_v_2.2/cacna1b	−1.11	−1.24	1.18	−1.01	1.07
Ca_v_2.3/cacna1e	1.02	−1.14	1.09	−1.01	1.26
Ca_v_3.1/cacna1g	1.04	−1.02	1.16	1.03	1.33
Ca_v_3.2/Cacna1h	1.02	−1.49	1.03	−1.06	1.53
Ca_v_3.3/cacna1i	−1.04	−1.16	−1.05	−1.18	1.25
Ca_v_ß1/cacnb1	−1.16	−1.51	1.04	−1.09	−1.11
Ca_v_ß2/cacnb2	1.10	−1.47	−1.04	−1.03	1.10
Ca_v_ß3/cacnb3	1.06	−1.11	1.01	−1.05	1.24
Ca_v_*α*2*δ*/cacna2d	1.01	−1.10	1.10	1.02	1.12
M_1/_Chrm1	1.75	1.10	−1.03	−1.09	1.01
M_3_/Chrm3	1.49	1.05	−1.12	−1.08	1.18
M_5/_Chrm5	1.42	−1.39	−1.01	−1.02	1.42

(B)	Motor Cortex (FC)				
	qPCR Groups				
Protein/gene	10 days	12 days	16 days	30 days	138 days

Ca_v_1.3/cacna1d	1.00	1.14	1.07	1.12	−1.35
Ca_v_2.1/cacna1a	−1.25	1.07	−1.27	1.01	1.01
Ca_v_2.2/cacna1b	−1.21	−1.05	1.00	1.03	−1.34
Ca_v_2.3/cacna1e	−1.13	−1.01	1.19	1.04	−1.26
Ca_v_3.1/cacna1g	−1.24	−1.04	1.04	−1.02	1.11
Ca_v_3.2/cacna1h	1.16	1.05	1.35	1.04	1.17
Ca_v_3.3/cacna1i	−1.13	−1.13	−1.02	−1.07	1.20
Ca_v_ß1/cacnb1	1.01	1.01	−1.09	−1.02	−1.01
Ca_v_ß2/cacnb2	1.23	1.01	1.32	1.04	−1.17
Ca_v_ß3/cacnb3	−1.11	1.04	−1.54	1.05	−1.11
Ca_v_*α*2*δ*/cacna2d	1.12	−1.02	1.13	1.09	1.29
M_1_/Chrm1	1.25	1.18	−1.23	1.05	1.02
M_3_/Chrm3	1.02	1.17	1.10	−1.07	1.19
M_5_/Chrm5	1.19	1.84	3.07	1.54	−1.26

Transcript fold changes (FC) were calculated based on RT-qPCR results of pilocarpine-treated rats and untreated controls using qBase^+^ software (Biogazelle, Belgium). As outlined previously, RNA was extracted at days 10, 12, 16, 30, and 138 pp (see also the illustration of the the triphasic injection regimen of the infant rat mTLE model in Figure 1 and Table 1).

## Data Availability

Data are available on request. Please contact the corresponding author Prof. Dr. Marco Weiergräber (MD, PhD); Experimental Neuropsychopharmacology, Federal Institute for Drugs and Medical Devices (Bundesinstitut für Arzneimittel und Medizinprodukte, BfArM), Kurt-Georg-Kiesinger-Allee 3, 53175 Bonn, Germany, email: marco.weiergraeber@bfarm.de.

## Supplementary Materials

Supplementary 1 Figure 1: electrode position for radiotelemetric EEG recordings.

Supplementary 2 Figure 2: body weight development in the InfRPil-mTLE model.

Supplementary 3 Figure 3: CA1 hippocampal electroencephalographic seizure analysis of the InfRPil-mTLE model using relative values.

Supplementary 4 Figure 4: effects of circadian rhythmicity (light/dark cycle) on CA1 hippocampal electroencephalographic seizure parameters in the InfRPil-mTLE model compared to controls.

Supplementary 5 Figure 5: M1 motor cortex electroencephalographic seizure analysis of the InfRPil-mTLE model using relative values.

Supplementary 6 Figure 6: effects of circadian rhythmicity (light/dark cycle) on M1 motor cortex electroencephalographic seizure parameters in the InfRPil-mTLE model compared to controls.

Supplementary 7 Table 1: overview of statistical results from hippocampal (CA1) seizure analysis in the InfRPil-mTLE model.

Supplementary 8 Table 2: overview of statistical results from motor cortex (M1) seizure analysis in the InfRPil-mTLE model.

Supplementary 9 Table 3: statistical overview of light (L) and dark (D) cycle effects on hippocampal (CA1) seizure parameters in pilocarpine-treated (P) and sham-treated control (C) rats.

Supplementary 10 Table 4: statistical overview of light (L) and dark (D) cycle effects on motor cortex (M1) seizure parameters in pilocarpine-treated (P) and sham-treated control (C) rats.

Supplementary 11 Table 5: statistical details of pilocarpine treatment on hippocampal (a) and cortical (b) transcript levels in the novel InfRPil-mTLE model compared to controls.
