# Supplementary material for: A Novel Rat Infant Model of Medial Temporal Lobe Epilepsy Reveals New Insight into the Molecular Biology and Epileptogenesis in the Developing Brain
Source: Neural Plast. 2024 Jul 25;2024:9946769. doi: 10.1155/2024/9946769 (PMC11300100; doi:10.1155/2024/9946769)
Supplement: Supplementary 1 — Figure 1: electrode position for radiotelemetric EEG recordings. [file 9946769.f1.pdf]

Supplementary figure 1 (Wormuth et al., 2024)

a

Wistar rats > 300 g; groups 2P/2C, 3P/3C;:AP: -3.8 mm, L: 2.5 mm; DV: 2.6 mm

CA1

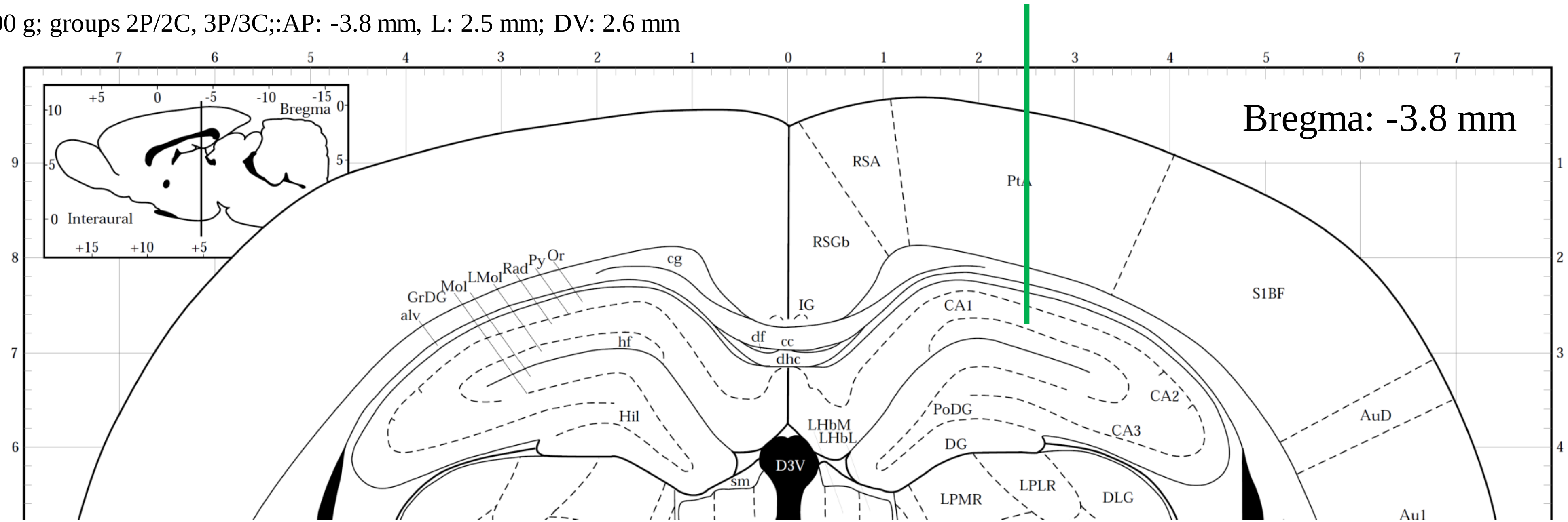

Wistar rats > 300 g; groups 2P/2C, 3P/3C;:AP: +2.5 mm, L: 3 mm; DV: 0 mm

M1

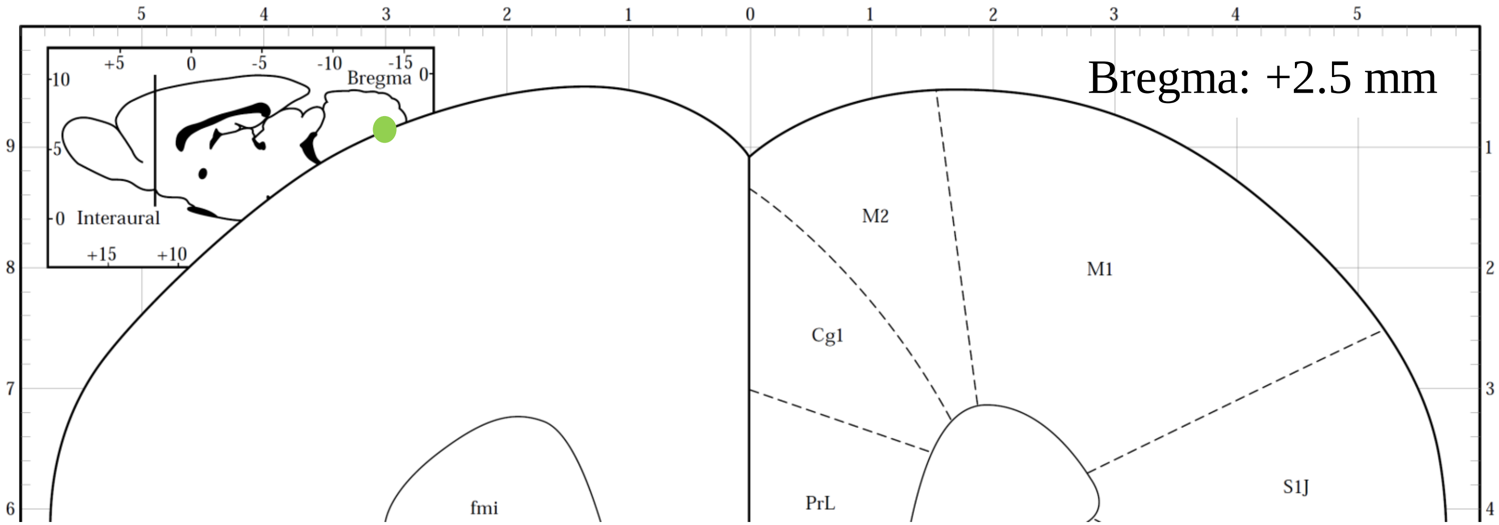

b

Wistar rats > 300 g; groups 2P/2C, 3P/3C;:AP: -3.8 mm, L: 2.5 mm; DV: 2.6 mm

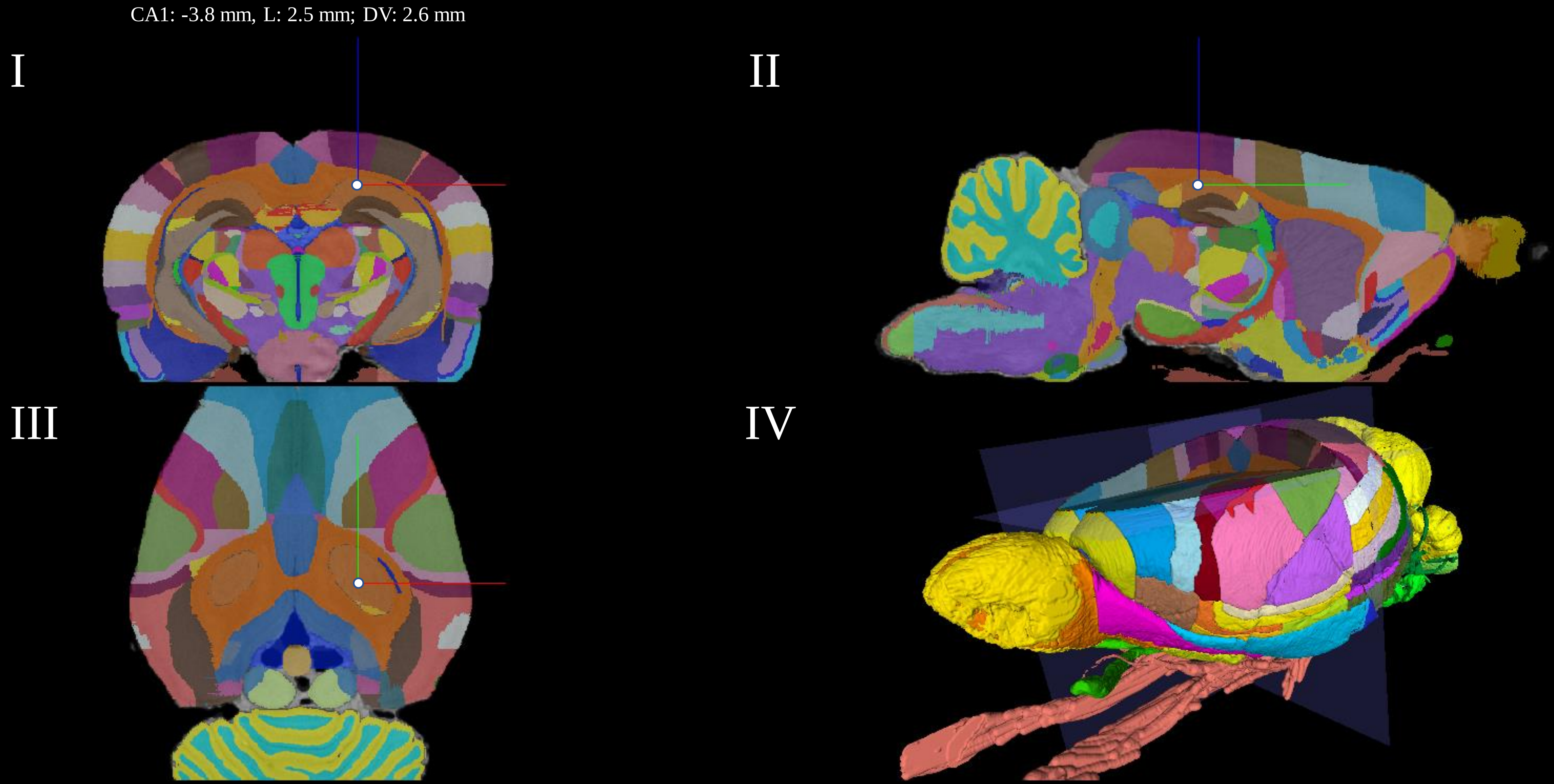

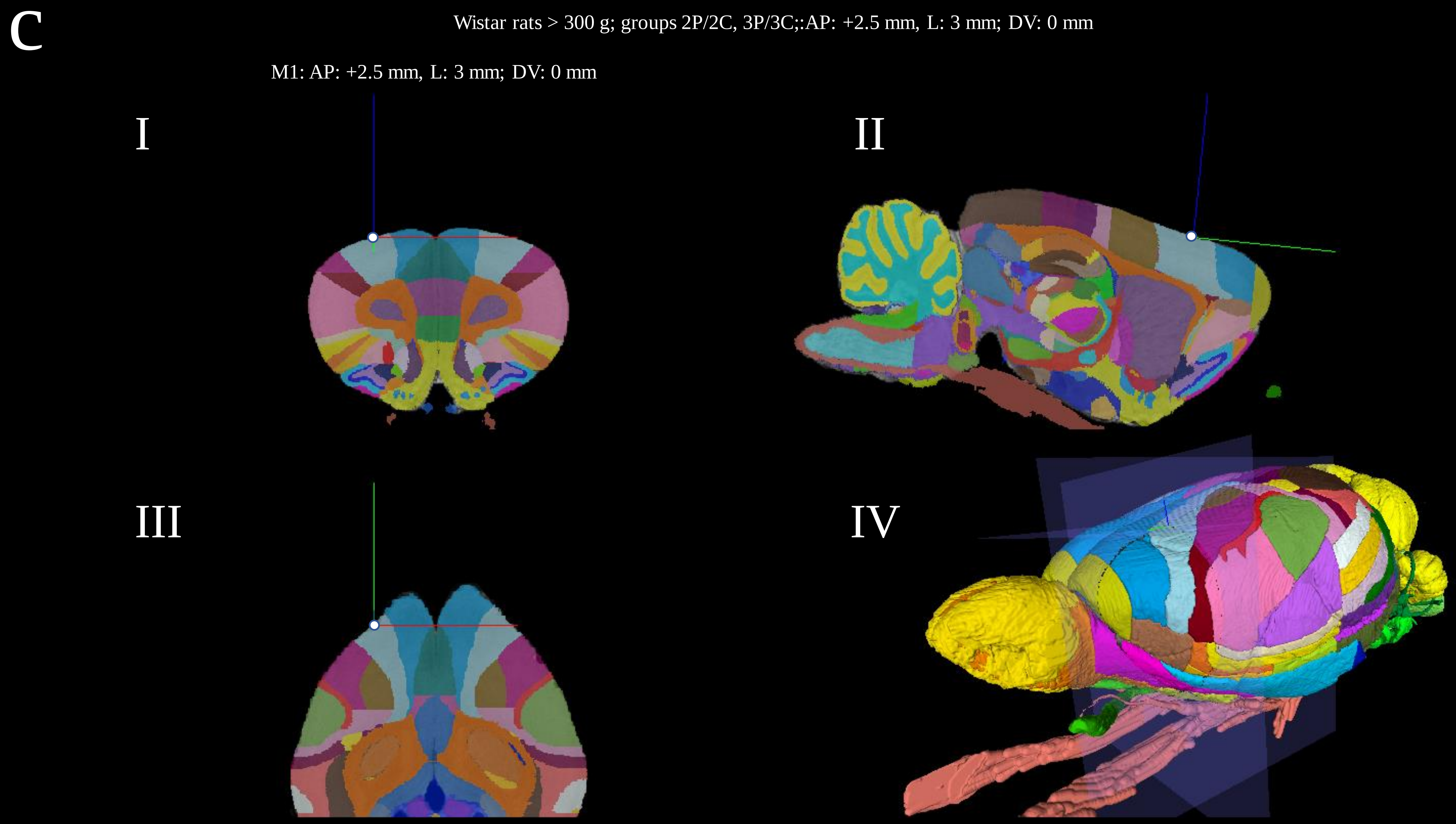
C
Wistar rats > 300 g; groups 2P/2C, 3P/3C;:AP: +2.5 mm, L: 3 mm; DV: 0 mm
M1: AP: +2.5 mm, L: 3 mm; DV: 0 mm
I
II
III
IV
