# Supplementary figures and images for: A Novel Rat Infant Model of Medial Temporal Lobe Epilepsy Reveals New Insight into the Molecular Biology and Epileptogenesis in the Developing Brain
Source: Neural Plast. 2024 Jul 25;2024:9946769. doi: 10.1155/2024/9946769 (PMC11300100; doi:10.1155/2024/9946769)

Supplementary figure 2 (Wormuth et al., 2024)

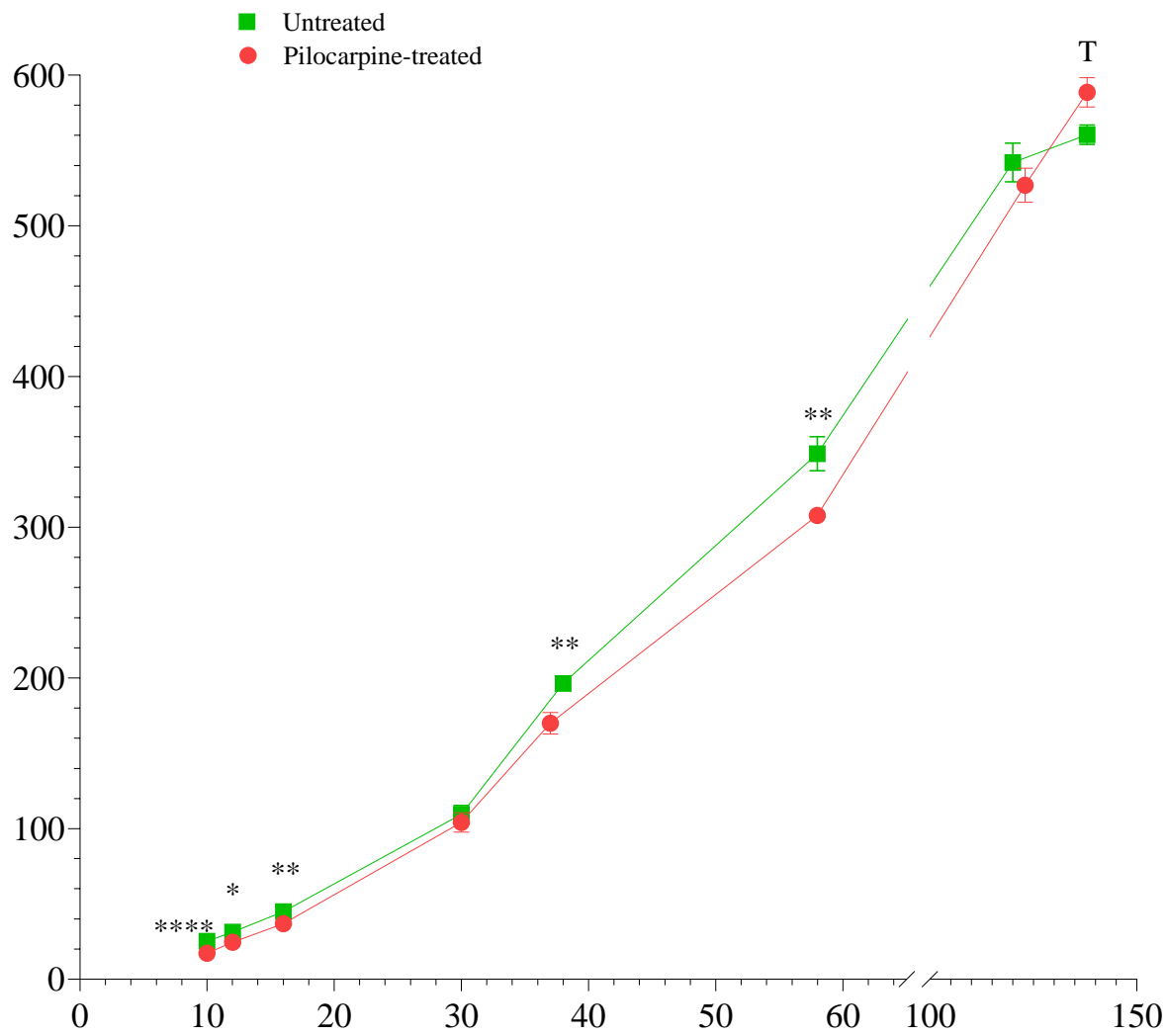

Supplement: Supplementary 2 — Figure 2: body weight development in the InfRPil-mTLE model. [file 9946769.f2.pdf]

# Hippocampus Seizure Analysis - Relative Values

A

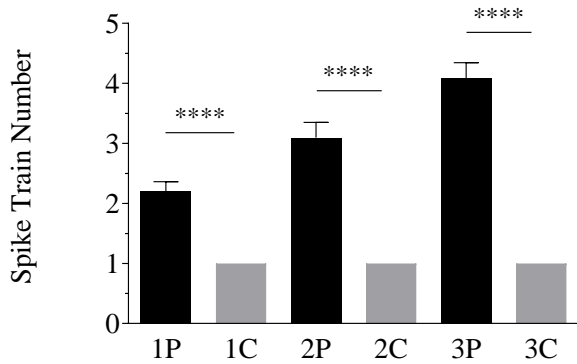

B

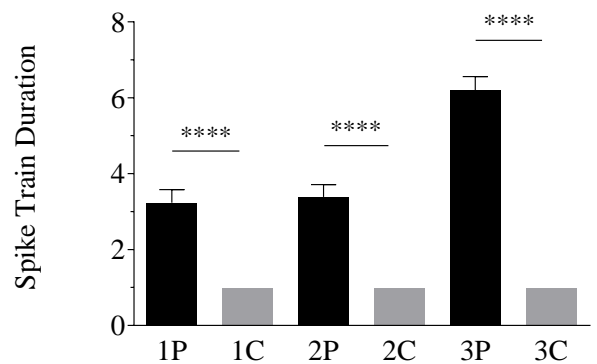

C

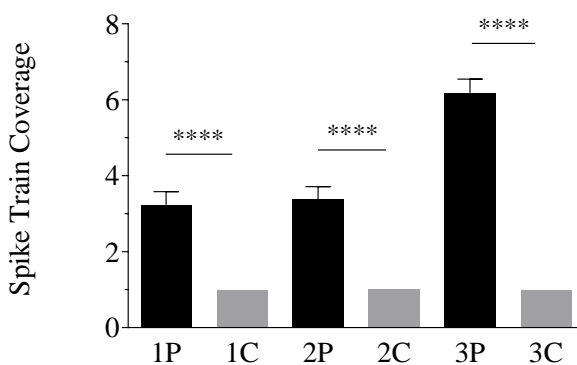

D

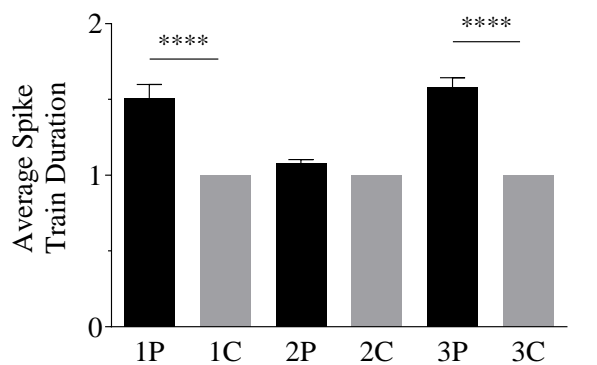

E

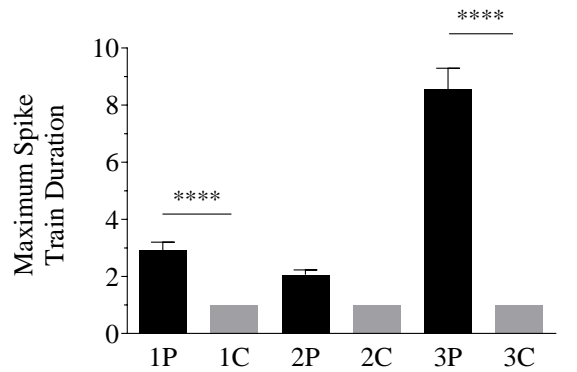

F

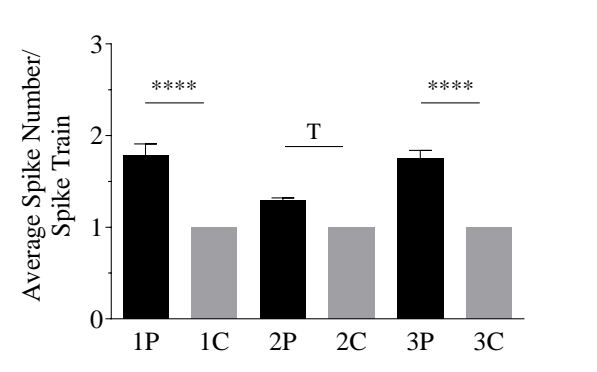

G

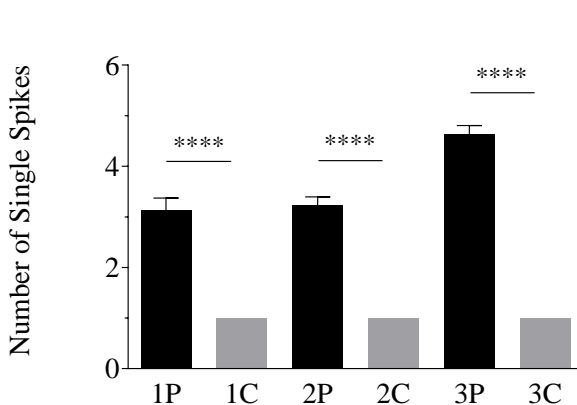

H

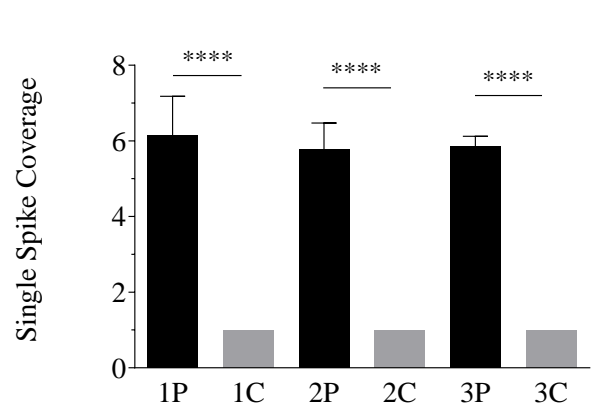

Supplement: Supplementary 3 — Figure 3: CA1 hippocampal electroencephalographic seizure analysis of the InfRPil-mTLE model using relative values. [file 9946769.f3.pdf]

# Hippocampus Dark & Light Seizure Analysis

A

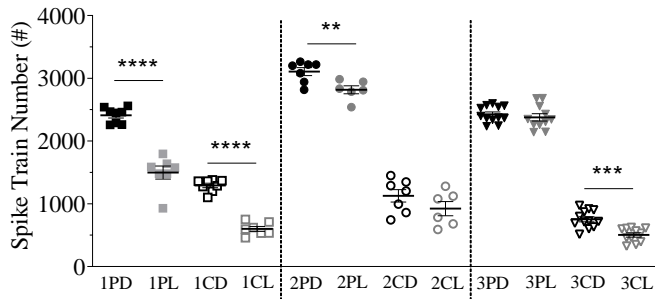

B

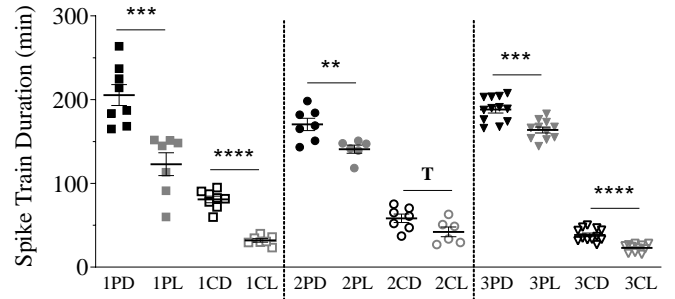

C

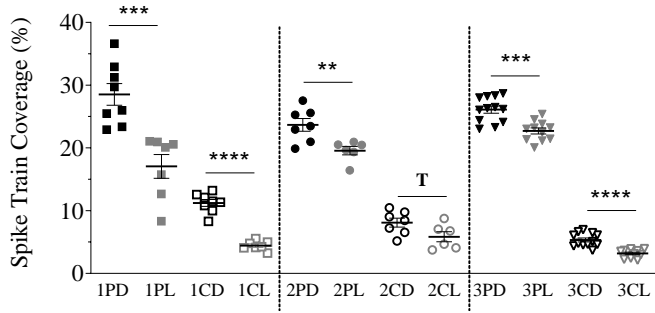

D

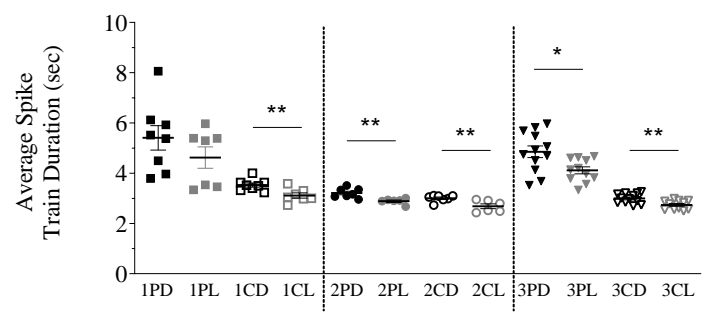

E

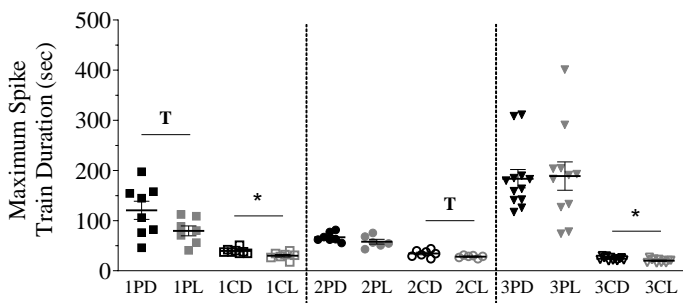

F

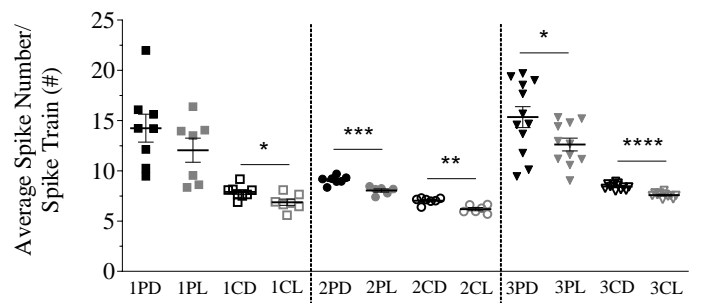

G

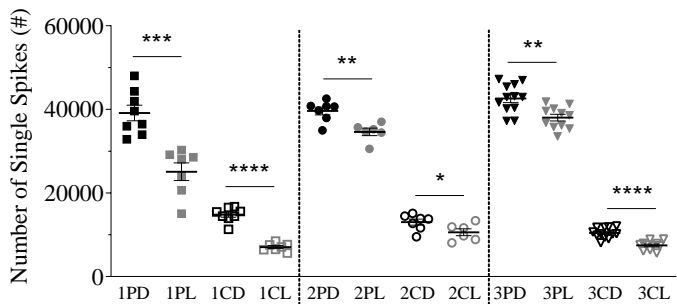

H

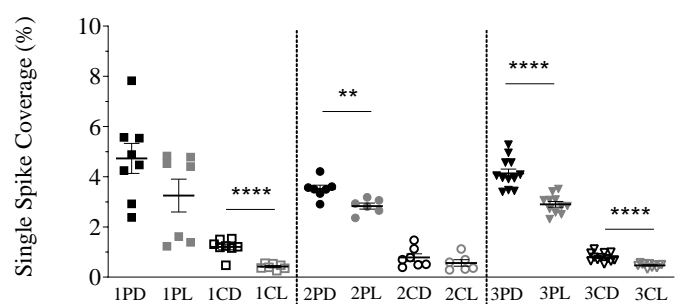

Supplement: Supplementary 4 — Figure 4: effects of circadian rhythmicity (light/dark cycle) on CA1 hippocampal electroencephalographic seizure parameters in the InfRPil-mTLE model compared to controls. [file 9946769.f4.pdf]

## Motor Cortex Seizure Analysis - Relative Values

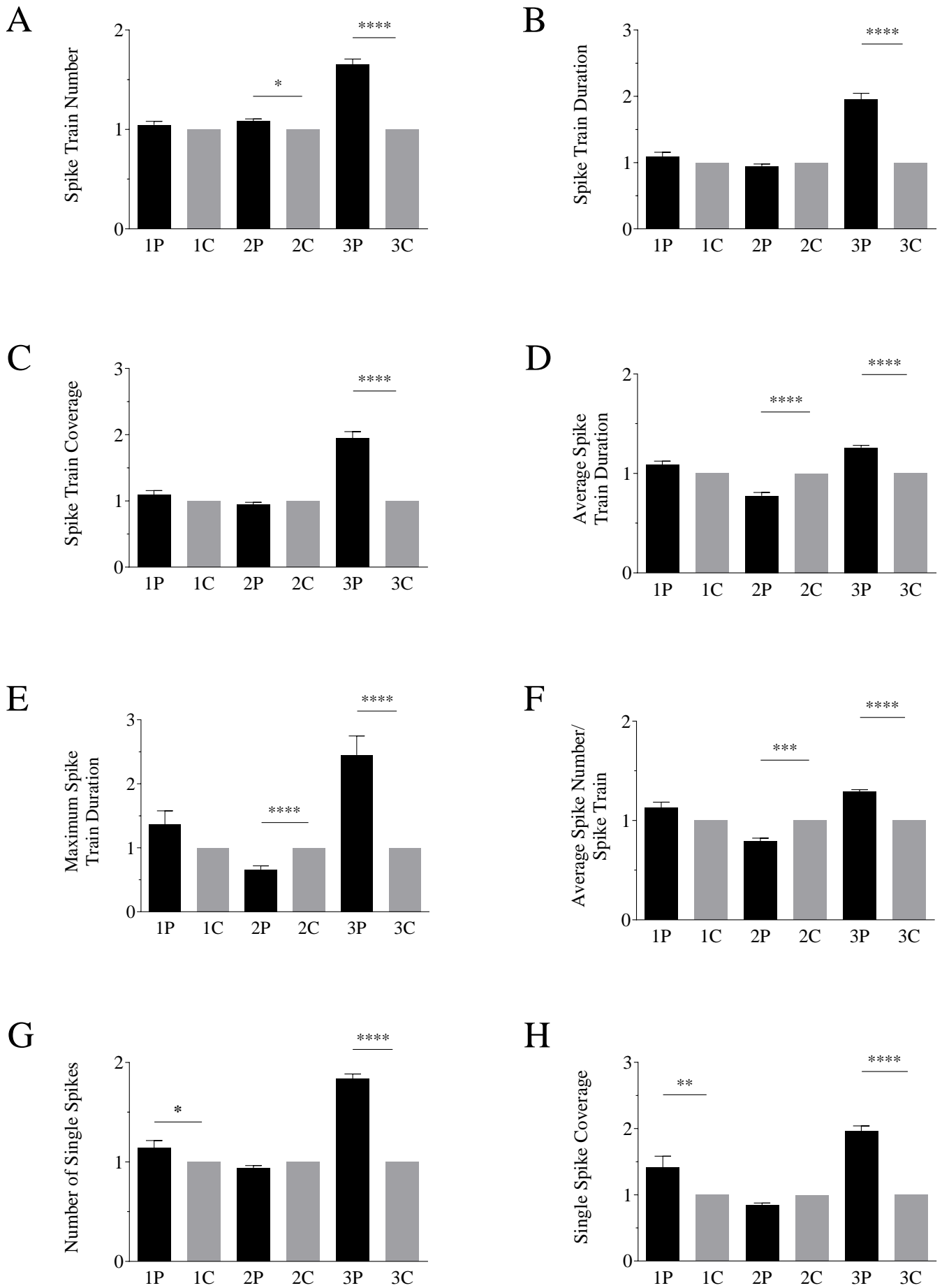

Supplement: Supplementary 5 — Figure 5: M1 motor cortex electroencephalographic seizure analysis of the InfRPil-mTLE model using relative values. [file 9946769.f5.pdf]

# Motor Cortex Dark & Light Seizure Analysis

A

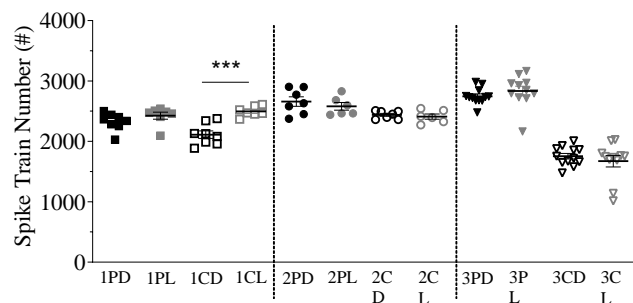

B

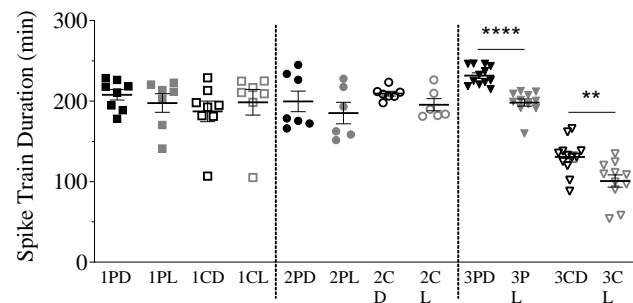

C

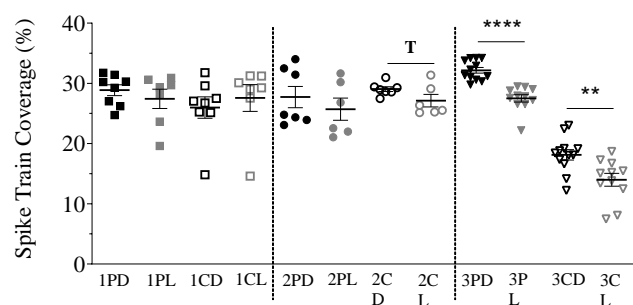

D

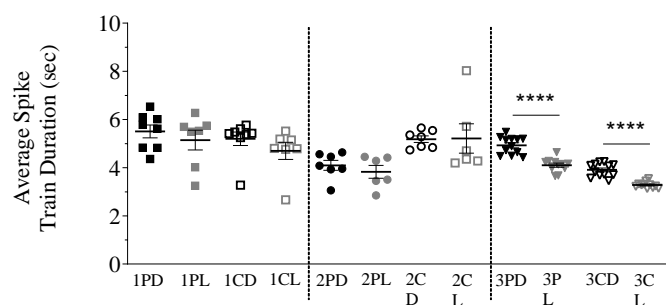

E

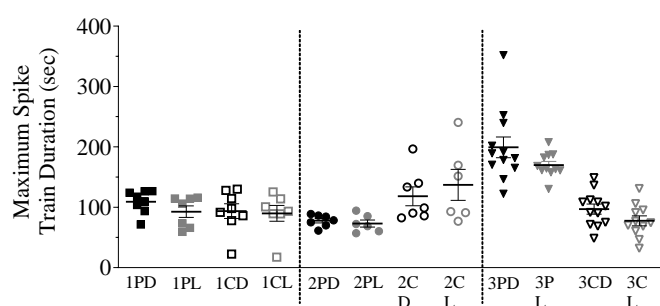

F

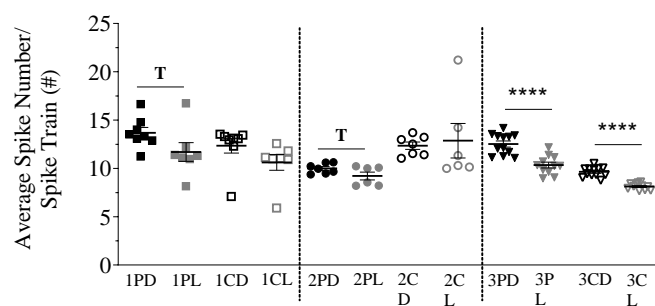

G

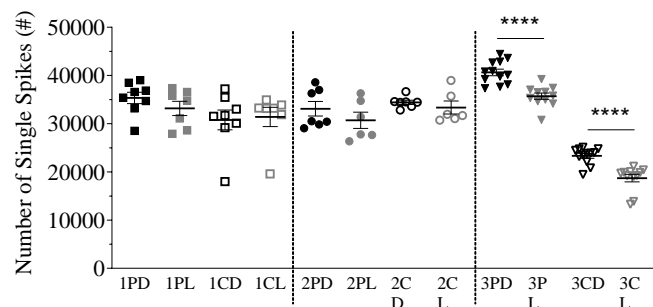

H

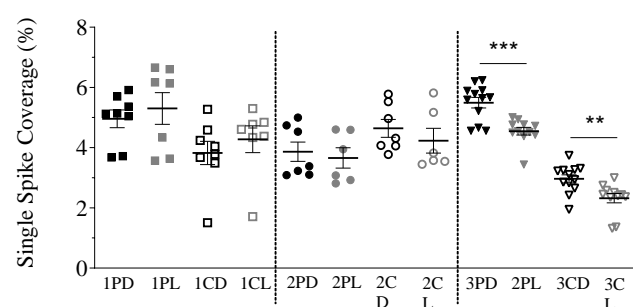

Supplement: Supplementary 6 — Figure 6: effects of circadian rhythmicity (light/dark cycle) on M1 motor cortex electroencephalographic seizure parameters in the InfRPil-mTLE model compared to controls. [file 9946769.f6.pdf]
