# Supplementary material for: A Novel Rat Infant Model of Medial Temporal Lobe Epilepsy Reveals New Insight into the Molecular Biology and Epileptogenesis in the Developing Brain
Source: Neural Plast. 2024 Jul 25;2024:9946769. doi: 10.1155/2024/9946769 (PMC11300100; doi:10.1155/2024/9946769)
Supplement: Supplementary 7 — Table 1: overview of statistical results from hippocampal (CA1) seizure analysis in the InfRPil-mTLE model. [file 9946769.f7.pdf]

Supplementary Table 1 (Wormuth et al., 2024)

Supplementary Table 1: Statistical analysis overview of hippocampal seizure parameters. Statistical comparison (red boxes) of the hippocampal seizure markers within the pilocarpine-treated (1P-3P) groups and within the sham-treated control (1C-3C) groups (one-way ANOVA), as well as between the pilocarpine-treated and their respective sham-treated control groups (1P/1C, 2P/2C, 3P/3C) (unpaired t-test).

Spike Train Number

Groups#	1P	1C	2P	2C	3P	3C
**1P**		**** ↓	**** ↑		*** ↑	
**1C**				ns		** ↓
**2P**				**** ↓	**** ↓	
**2C**						*** ↓
**3P**						**** ↓
**3C**						

Spike Train Duration

Groups#	1P	1C	2P	2C	3P	3C
**1P**		**** ↓	ns		ns	
**1C**				ns		*** ↓
**2P**				**** ↓	ns	
**2C**						** ↓
**3P**						**** ↓
**3C**						

Spike Train Coverage

Groups#	1P	1C	2P	2C	3P	3C
**1P**		**** ↓	ns		ns	
**1C**				ns		*** ↓
**2P**				**** ↓	ns	
**2C**						** ↓
**3P**						**** ↓
**3C**						

Average Spike Train Duration

Groups#	1P	1C	2P	2C	3P	3C
**1P**		**** ↓	**** ↓		ns	
**1C**				**** ↓		**** ↓
**2P**				* ↓	**** ↑	
**2C**						ns
**3P**						**** ↓
**3C**						

Maximum Spike Train Duration

Groups#	1P	1C	2P	2C	3P	3C
**1P**		**** ↓	ns		*** ↑	
**1C**				ns		**** ↓
**2P**				**** ↓	**** ↑	
**2C**						**** ↓
**3P**						**** ↓
**3C**						

Average Spike Count/Spike Train

#Groups	1P	1C	2P	2C	3P	3C
**1P**		**** ↓	*** ↓		ns	
**1C**				** ↓		* ↑
**2P**				**** ↓	**** ↑	
**2C**						**** ↑
**3P**						**** ↓
**3C**						

Single Spike Count

Groups#	1P	1C	2P	2C	3P	3C
**1P**		**** ↓	↗		*** ↑	
**1C**				ns		↗
**2P**				**** ↓	ns	
**2C**						* ↓
**3P**						**** ↓
**3C**						

Single Spike Coverage

Groups#	1P	1C	2P	2C	3P	3C
**1P**		**** ↓	ns		ns	
**1C**				ns		ns
**2P**				**** ↓	ns	
**2C**						ns
**3P**						**** ↓
**3C**						

Pilocarpine-treated groups (1P-3P) were injected with pilocarpine (180 mg/kg) at days 9, 11, 15 pp according to the triphasic pilocarpine injection regime new rat mTLE model descripted in **Fig. 1.** Sham-treated control groups (1C-3C) received NaCl 0.9% instead of pilocarpine. Groups 1P and 1C correspond to animals that were implanted at days 36-38 pp and continuously recorded on days 43-50 pp and respectively, groups 2P and 2C at days 57-59 pp and on days 64-70 pp and groups 3P and 3C at days 119-123 and on days 127-134 & 137-142 pp. (*, $p \leq 0.05$; **, $p \leq 0.01$; ***, $p \leq 0.001$; ****, $p \leq 0.0001$; $0.05 < p < 0.1$, statistical trend; ns, $p \geq 0.1$ not significant; ↑, significant higher; ↓, significant lower; ↗, statistical trend).
