# Supplementary material for: A Novel Rat Infant Model of Medial Temporal Lobe Epilepsy Reveals New Insight into the Molecular Biology and Epileptogenesis in the Developing Brain
Source: Neural Plast. 2024 Jul 25;2024:9946769. doi: 10.1155/2024/9946769 (PMC11300100; doi:10.1155/2024/9946769)
Supplement: Supplementary 9 — Table 3: statistical overview of light (L) and dark (D) cycle effects on hippocampal (CA1) seizure parameters in pilocarpine-treated (P) and sham-treated control (C) rats. [file 9946769.f9.pdf]

Supplementary table 3: Statistical overview of light (L) and dark (D) effects on hippocampal seizure parameters in pilocarpine-treated (P) and sham-treated control rats.

	Seizure Markers							
	Spike Train Number	Spike Train Duration	Spike Train Coverage	Average Spike Train Duration	Maximum Spike Train Duration	Average Spike Number per Spike Train	Number of Single Spikes	Single Spike Coverage
1P D vs. 1P L	****	*** 0.0006	*** 0.0006	ns	T 0.0796	ns	*** 0.0002	ns
1C D vs. 1C L	****	****	****	** 0.0073	* 0.0108	* 0.0186	****	****
2P D vs. 2P L	** 0.0086	** 0.0078	** 0.0078	** 0.0040	ns	*** 0.0007	** 0.0027	** 0.0046
2C D vs. 2C L	ns	T 0.0597	T 0.0595	** 0.0097	T 0.0581	** 0.0011	* 0.0481	ns
3P D vs. 3P L	ns	*** 0.0002	*** 0.0002	* 0.0144	ns	* 0.0405	** 0.0019	****
3C D vs. 3C L	*** 0.0001	****	****	** 0.0030	* 0.0138	****	****	****
1P D vs. 1C D	****	****	****	** 0.0020	*** 0.0005	*** 0.0005	****	****
1P L vs. 1C L	****	****	****	** 0.0048	*** 0.0004	** 0.0013	****	*** 0.0010
2P D vs. 2C D	****	****	****	* 0.0362	****	****	****	****
2P L vs. 2C L	****	****	****	T 0.0813	*** 0.0001	****	****	****
3P D vs. 3C D	****	****	****	****	****	****	****	****
3P L vs. 3C L	****	****	****	****	****	****	****	****
1P D vs. 2P D	****	* 0.0363	* 0.0363	** 0.0011	* 0.0175	** 0.0044	ns	T 0.0883
1P D vs. 3P D	ns	ns	ns	ns	* 0.0308	ns	T 0.0909	ns
2P D vs. 3P D	****	* 0.0359	* 0.0373	****	*** 0.0002	*** 0.0003	T 0.0608	* 0.0275
1P L vs. 2P L	****	ns	ns	** 0.0033	T 0.0914	* 0.0108	** 0.0024	ns
1P L vs. 3P L	****	** 0.0028	** 0.0028	ns	** 0.0090	ns	****	ns
2P L vs. 3P L	*** 0.0002	** 0.0015	** 0.0016	****	** 0.0044	*** 0.0001	* 0.0173	ns
1C D vs. 2C D	ns	** 0.0036	** 0.0036	*** 0.0002	ns	** 0.0077	T 0.0815	* 0.0406
1C D vs. 3C D	****	****	****	****	****	* 0.0231	****	** 0.0020
2C D vs. 3C D	*** 0.0009	*** 0.0007	*** 0.0007	ns	*** 0.0004	****	** 0.0024	ns
1C L vs. 2C L	* 0.0150	ns	ns	* 0.0109	ns	T 0.0885	** 0.0016	ns
1C L vs. 3C L	T 0.0815	** 0.0020	** 0.0020	** 0.0025	** 0.0010	* 0.0154	ns	ns
2C L vs. 3C L	*** 0.0004	*** 0.0009	*** 0.0009	ns	*** 0.0003	****	*** 0.0007	ns
