# Supplementary material for: A Novel Rat Infant Model of Medial Temporal Lobe Epilepsy Reveals New Insight into the Molecular Biology and Epileptogenesis in the Developing Brain
Source: Neural Plast. 2024 Jul 25;2024:9946769. doi: 10.1155/2024/9946769 (PMC11300100; doi:10.1155/2024/9946769)
Supplement: Supplementary 10 — Table 4: statistical overview of light (L) and dark (D) cycle effects on motor cortex (M1) seizure parameters in pilocarpine-treated (P) and sham-treated control (C) rats. [file 9946769.f10.pdf]

Supplementary table 4: Statistical overview of light (L) and dark (D) effects on motor cortex (M1) seizure parameters in pilocarpine-treated (P) and sham-treated control rats.

	Spike Train Number	Spike Train Duration	Spike Train Coverage	Average Spike Train Duration	Maximum Spike Train Duration	Average Spike Number per Spike Train	Number of Single Spikes	Single Spike Coverage
1P D vs. 1P L	ns	ns	ns	ns	ns	T 0.0887	ns	ns
1C D vs. 1C L	*** 0.0001	ns	ns	ns	ns	ns	ns	ns
2P D vs. 2P L	ns	ns	ns	ns	ns	T 0.0952	ns	ns
2C D vs. 2C L	ns	T 0.0979	T 0.0954	ns	ns	ns	ns	ns
3P D vs. 3P L	ns	****	****	****	ns	****	****	*** 0.0003
3C D vs. 3C L	ns	** 0.0061	** 0.0061	****	ns	****	****	** 0.0054
1P D vs. 1C D	* 0.0179	ns	ns	ns	ns	ns	T 0.0747	* 0.0353
1P L vs. 1C L	ns	ns	ns	ns	ns	ns	ns	ns
2P D vs. 2C D	* 0.0207	ns	ns	*** 0.0009	* 0.0275	*** 0.0001	ns	ns
2P L vs. 2C L	T 0.0513	ns	ns	T 0.0636	* 0.0347	T 0.0745	ns	ns
3P D vs. 3C D	****	****	****	****	****	****	****	****
3P L vs. 3C L	****	****	****	****	****	****	****	****
1P D vs. 2P D	** 0.0031	ns	ns	** 0.0012	** 0.0019	****	ns	* 0.0261
1P D vs. 3P D	****	**0.0025	**0.0025	* 0.0329	*** 0.0007	T 0.0644	*** 0.0006	ns
2P D vs. 3P D	ns	** 0.0075	** 0.0077	*** 0.0009	****	****	****	*** 0.0001
1P L vs. 2P L	T 0.0983	ns	ns	* 0.0254	ns	* 0.0475	ns	* 0.0286
1P L vs. 3P L	** 0.0021	ns	ns	** 0.0073	****	ns	T 0.0952	ns
2P L vs. 3P L	T 0.0507	ns	ns	ns	****	* 0.0374	** 0.0047	** 0.0095
1C D vs. 2C D	*** 0.0004	ns	ns	ns	ns	ns	ns	ns
1C D vs. 3C D	*** 0.0002	*** 0.0003	*** 0.0003	****	ns	*** 0.0005	*** 0.0005	* 0.0257
2C D vs. 3C D	****	****	****	****	ns	****	****	****
1C L vs. 2C L	ns	ns	ns	ns	ns	ns	ns	ns
1C L vs. 3C L	****	****	****	*** 0.0001	ns	** 0.0015	****	*** 0.0002
2C L vs. 3C L	****	****	****	*** 0.0005	* 0.0148	** 0.0023	****	*** 0.0001
