# Supplementary material for: A Novel Rat Infant Model of Medial Temporal Lobe Epilepsy Reveals New Insight into the Molecular Biology and Epileptogenesis in the Developing Brain
Source: Neural Plast. 2024 Jul 25;2024:9946769. doi: 10.1155/2024/9946769 (PMC11300100; doi:10.1155/2024/9946769)
Supplement: Supplementary 11 — Table 5: statistical details of pilocarpine treatment on hippocampal (a) and cortical (b) transcript levels in the novel InfRPil-mTLE model compared to controls. [file 9946769.f11.pdf]

Supplementary table 5: Overall effect of pilocarpine treatment on the hippocampal (A) and cortical (B) gene expression in infantile rats.
All animals treated with pilocarpine were compared with all untreated animals independently of their age, or the number of pilocarpine injections that they received. Hippocampal and cortical RNA was extracted at days 10, 12, 16, 30 and 138 pp (corresponding to groups 10d, 12d, 16d, 30d and 138d respectively) from neonatal rat pups treated according to the new neonatal triphasic injection regime mTLE rat model (**Fig. 1**). RNA was used to identify the pilocarpine effect on the transcriptome profile of VGCC ($Ca_v1.3$, $Ca_v2.1$, $Ca_v2.2$, $Ca_v2.3$, $Ca_v3.1$, $Ca_v3.2$, $Ca_v3.3$), VGCC auxiliary subunits (β_1, β_2, β_3, $\alpha_2\delta$) and muscarinic receptors (M_1, M_2, M_3) by RT-qPCR. Based on the new neonatal triphasic injection regime mTLE rat model, pilocarpine-treated animals of group 10d received one pilocarpine injection (170 mg/kg) at day 9 pp; group 12d received two pilocarpine injections at days 9 and 11 pp; groups 16d, 30d and 18d received three pilocarpine injections at days 9, 11 and 15 pp. Control animals received no treatment. Statistical comparison (one-way ANOVA) between all pilocarpine-treated animals to all untreated control animals from all above mentioned groups. (*, $p \leq 0.05$; **, $p \leq 0.01$; ***, $p \leq 0.001$; ****).

A) Hippocampus

	$Ca_v1.3$	$Ca_v2.1$	$Ca_v2.2$	$Ca_v2.3$	$Ca_v3.1$	$Ca_v3.2$	$Ca_v3.3$	$Ca_v\beta1$	$Ca_v\beta2$	$Ca_v\beta3$	$Ca_v\alpha2\delta$	Chrm1	Chrm3	Chrm5
Treated vs Untreated	p = 2.872E-3	p = 1.944E-3	p = 7.623E-10	p = 2.489E-6	p = 1.625E-9	p = 1.065E-8	p = 9.007E-8	p = 4.389E-8	p = 2.204E-13	p = 1.927E-8	p = 1.278E-7	p = 3.875E-8	p = 3.481E-4	p = 4.318E-3
	**	**	****	****	****	****	****	****	****	****	****	****	***	**

B) Motor cortex

	$Ca_v1.3$	$Ca_v2.1$	$Ca_v2.2$	$Ca_v2.3$	$Ca_v3.1$	$Ca_v3.2$	$Ca_v3.3$	$Ca_v\beta1$	$Ca_v\beta2$	$Ca_v\beta3$	$Ca_v\alpha2\delta$	Chrm1	Chrm3	Chrm5
Treated vs Untreated	p = 6.765E-6	p = 2.343E-7	p = 1.187E-7	p = 2.897E-6	p = 3.212E-5	p = 3.971E-2	p = 4.228E-6	p = 1.663E-4	p = 8.704E-9	p = 2.567E-7	p = 6.497E-7	p = 4.982E-2	p = 2.474E-4	p = 5.732E-5
	****	****	****	****	****	*	****	***	****	****	****	*	***	****
